# Supplementary material for: Implementing online consultations in primary care: a mixed-method evaluation extending normalisation process theory through service co-production
Source: BMJ Open. 2018 Mar 19;8(3):e019966. doi: 10.1136/bmjopen-2017-019966 (PMC5875620; doi:10.1136/bmjopen-2017-019966)
Supplement: Supplementary file 1 [file bmjopen-2017-019966supp001.pdf]

SUPPLEMENTARY FILE

Table A: Patient survey – how and why accessed e-consultations, and resulting satisfaction

Some of these questions are expanded further with free-text responses, overviewed in Table B.

	% (n)
How did you hear about the Consult Online from Home Service?	
My GP told me about it	0.3% (2)
Someone else from the GP practice	4.2% (33)
From the GP practice website	58.0% (455)
Another patient/family member	4.2% (33)
From an internet search	5.6% (44)
I read about it	2.7% (21)
From a leaflet or promotional banner	18.5% (145)
Other → Free text	6.6% (52)
When your practice contacted you about your Consult Online From Home Service assessment, what were you advised to do?	
How to look after the problem myself, without contacting the GP practice/other health service	9.1% (56)
Pick up a prescription from the surgery /pharmacy	27.6% (169)
Visit the GP practice for face-to-face at later date	40.0% (244)
Go to A&E department / Walk-in Centre etc	1.5% (9)
Was not contacted	13.7% (84)
Other → Free text	8.3% (51)
How likely are you to recommend the Consult Online From Home Service to friends and family if they need similar care or advice?	
Extremely likely	55.5% (422)
Likely	25.2% (192)
Neither likely nor unlikely	7.2% (55)
Unlikely	6.6% (50)
Extremely unlikely	5.5% (42)
Would you use the service again instead of booking a face to face appointment?	
Yes	76.3% (582)
No	8.5% (65)
Not sure	15.2% (116)
Thinking about your use of the Consult Online From Home Service, would you agree or disagree with the statements below<br>The Consult Online From Home Service was easy to use.	
Strongly agree	50.7% (383)
Agree	37.6% (284)
Neither agree nor disagree	5.2% (39)
Disagree	5.2% (39)
Strongly disagree	1.5% (11)

	% (n)
Overall, how satisfied or dissatisfied were you with using the Consult Online From Home Service for your health assessment?	
Very satisfied	60.3% (459)
Fairly satisfied	20.4% (155)
Neither satisfied nor dissatisfied	4.3% (33)
Fairly dissatisfied	7.4% (56)
Very dissatisfied	7.6% (58)

Table B: E-consultation patient survey free text response numbers

Table B summarises the free text questions from the eConsult online patient satisfaction survey, with response numbers. Average word counts of comments, alongside the range of comments from the lowest number of words in a comment to the highest are provided to give details on the breadth and depth of comments

E-consultation survey question	Number of free text responses	Average word count of comments	Word count range of comments
How did you hear about the Consult Online?	49	6.3	2 to 18
If the Consult Online service had not been available, what would you have done about your health problem?	37	11.4	1 to 67
Was there a practical reason why you used the Consult Online?	182	12.9	1 to 68
When your practice contacted you about your Consult Online assessment, what were you advised to do?	224	10.8	1 to 93
Did you follow the Consult Online advice? (if answered no, reason why they did not follow advice)	74	8.6	1 to 27
Did any of our staff make your experience particularly good?	343	16.7	1 to 187
Overall, how satisfied or dissatisfied were you with using the Consult Online Service for your health assessment? Reason for this	510	28.1	1 to 257
What improvements would you make to the service?	512	16.3	1 to 127
