# Supplementary material for: Implementing online consultations in primary care: a mixed-method evaluation extending normalisation process theory through service co-production
Source: BMJ Open. 2018 Mar 19;8(3):e019966. doi: 10.1136/bmjopen-2017-019966 (PMC5875620; doi:10.1136/bmjopen-2017-019966)
Supplement: Author's manuscript [file bmjopen-2017-019966.draft_revisions.pdf]

BMJ Open

BMJ Open is committed to open peer review. As part of this commitment we make the peer review history of every article we publish publicly available.

When an article is published we post the peer reviewers' comments and the authors' responses online. We also post the versions of the paper that were used during peer review. These are the versions that the peer review comments apply to.

The versions of the paper that follow are the versions that were submitted during the peer review process. They are not the versions of record or the final published versions. They should not be cited or distributed as the published version of this manuscript.

BMJ Open is an open access journal and the full, final, typeset and author-corrected version of record of the manuscript is available on our site with no access controls, subscription charges or pay-per-view fees (http://bmjopen.bmj.com).

If you have any questions on BMJ Open's open peer review process please email editorial.bmjopen@bmj.com

BMJ Open

Implementing online consultations in primary care: A mixed method evaluation extending normalisation process theory through service co-production

Journal:	*BMJ Open*
Manuscript ID	bmjopen-2017-019966
Article Type:	Research
Date Submitted by the Author:	09-Oct-2017

Complete List of Authors:	Farr, Michelle; University of Bristol, Population Health Sciences, Bristol Medical School; NIHR CLAHRC West, University Hospitals Bristol NHS Foundation Trust, 9th Floor, Whitefriars, Lewins Mead<br>Banks, Jonathan; University of Bristol, Population Health Sciences, Bristol Medical School; NIHR CLAHRC West, , University Hospitals Bristol NHS Foundation Trust<br>Edwards, Hannah; University of Bristol, Population Health Sciences, Bristol Medical School; NIHR CLAHRC West, University Hospitals Bristol NHS Foundation Trust<br>Northstone, Kate; University of Bristol, Population Health Sciences, Bristol Medical School; ALSPAC, University of Bristol<br>Bernard, Elly; One Care (BNSSG) Ltd<br>Salisbury, Chris; University of Bristol, Population Health Sciences, Bristol Medical School; NIHR CLAHRC West, University Hospitals Bristol NHS Foundation Trust<br>Horwood, Jeremy; University of Bristol, UK, Population Health Sciences, Bristol Medical School; NIHR CLAHRC West, University Hospitals Bristol NHS Foundation Trust

**Primary Subject Heading**:	General practice / Family practice
Secondary Subject Heading:	Health informatics
Keywords:	Co-production, Telemedicine < BIOTECHNOLOGY & BIOINFORMATICS, PRIMARY CARE, normalisation process theory, online consultations, touchpoints

SCHOLARONE™ Manuscripts

Implementing online consultations in primary care: A mixed method evaluation extending normalisation process theory through service co-production

Research article

Authors

Michelle Farr[1, 2] M.Farr@bristol.ac.uk
Jon Banks[1, 2] Jon.Banks@bristol.ac.uk
Hannah Edwards[1, 2] Hannah.Edwards@bristol.ac.uk
Kate Northstone[1, 2] Kate.Northstone@bristol.ac.uk
Elly Bernard[3] elly.bernard@onecare.org.uk
Chris Salisbury[1, 2] C.Salisbury@bristol.ac.uk
Jeremy Horwood[1, 2] J.Horwood@bristol.ac.uk

1. Population Health Sciences, Bristol Medical School, University of Bristol, Canynge Hall, 39 Whatley Road, Bristol, BS8 2PS, UK.

2. National Institute for Health Research Collaboration for Leadership in Applied Health Research and Care (NIHR CLAHRC) West at University Hospitals Bristol NHS Foundation Trust, 9th Floor, Whitefriars, Lewins Mead, Bristol, BS1 2NT

3. One Care (BNSSG) Ltd, Unit 4 Osprey Court, Hawkfield Business Park, Whitchurch, Bristol, BS14 0BB.

Corresponding Author:

Michelle Farr
NIHR CLAHRC West
9th Floor Whitefriars
Lewins Mead
Bristol BS1 2NT
M.Farr@bristol.ac.uk
0117 342 7279

Word count:

3,995

For peer review only

ABSTRACT

Objectives: An evaluation of implementing an online consultation system into UK primary care practices. We describe how e-consultations changed the way that general practice (GP) services were produced and experienced, and with what consequences, for patients and GPs.

Design: Mixed method evaluation of a primary care e-consultation system.

Setting: Primary care practices in south-west England.

Methods: Qualitative interviews with 23 practice staff in 6 practices. Patient survey data for 756 e-consultations from 36 practices, with free text survey comments from 512 patients, were analysed thematically. Anonymised patients' records were abstracted for 485 e-consultations from 8 practices, including consultation types and outcomes. Statistical analysis included descriptive statistics, chi-square tests or odds ratios with 95% confidence intervals and p-values. Analysis of implementation and usage of the e-consultation system was informed by: (a) normalisation process theory, (b) a process map that illustrates how e-consultations were co-produced, and (c) patients' and staff touchpoints.

Results: We found different expectations between patients and staff on how to use e-consultations 'appropriately'. Whilst some patients used the system to try and save time for themselves and their GPs, some used e-consultations when they could not get a timely face-to-face appointment. Most e-consultations resulted in either follow-on phone (32%) or face-to-face appointments (38%) and GPs felt that this duplicated their workload. Patient satisfaction of the system was high, but a minority were dissatisfied with practice communication about their e-consultation.

Conclusions: Where both patients and staff interact with technology, it is in effect 'co-implemented'. How patients used e-consultations impacted upon practice staff's experiences and appraisal of the system. Overall, e-consultations were not an immediate solution for efficiency savings, but could improve access for some patients. Mapping the co-production of the e-consultation process through touchpoints provides an analytic lens to understand how the e-consultation system and its implementation can be improved.

Keywords

Co-production, telemedicine, normalisation process theory, online consultations, primary care, touchpoints

For peer review only - http://bmjopen.bmj.com/site/about/guidelines.xhtml

Strengths and limitations of this study

- Largest UK study to date examining staff and patient experiences of using a primary care online consultation system.
- Extending normalisation process theory with service co-production theory enables an in-depth understanding of how patients and staff interacted with the e-consultation system.
- Touchpoint analysis enables improvements to be suggested to develop the design and implementation of online consultation systems, aimed at software designers, policymakers and general practices interested in this technology.
- This observational study was based on a pilot period of one online consultation system; and issues highlighted may be due to the system studied, rather than all online consultation systems.

BACKGROUND

English general practice clinical workload has risen by 16% over the period 2007-14.[1] 93% of general practitioners (GPs) say their workload has negatively impacted on quality of care given to patients.[2] Average waiting times for an appointment have been reported as just under 13 days.[3] Internationally, policymakers are advocating technological alternatives to face-to-face primary care consultations to improve service quality.[4] UK policy to improve primary care access includes the Prime Minister's Challenge Fund (now the GP Access Fund),[5] and the General Practice Forward View.[6] These promote greater use of technology to manage workload and improve patient access, with £45 million made available to support the implementation of online consultation systems.[7] Online or e-consultations enable patients to contact their GP via a mobile app or online portal.[7] General practice staff attitudes toward electronically based consultations include concerns about medico-legal issues, clinical limitations and increasing workload concerns.[8-10] Research into practitioners' and patients' actual experiences of e-consultations is limited, but timely, as implementation is at an early stage.[4 10]

A consortium of general practices in south west England [One Care],[11] received funding through the GP Access Fund,[5] to pilot online consultations. Starting in April 2015, the eConsult system[12] (previously called WebGP) was implemented free of charge into 36 general practices. Patients access the eConsult system (referred to as 'the system' in this paper) via their own GP practice website. They can access self-help, pharmacy advice, 111 (NHS non-emergency telephone advice), administrative help (such as repeat prescriptions), or submit an online form with details of their condition, electronically sending this to their GP practice, where it is then processed. If the system identifies signs or symptoms that may require immediate medical attention, patients are redirected

For peer review only - http://bmjopen.bmj.com/site/about/guidelines.xhtml

to appropriate services, otherwise the system informs patients that their GP practice will contact them by the end of the next working day.

Normalisation process theory (NPT) illustrates issues to address when implementing a technology or complex intervention (Table 1).[13-15]

Table 1: NPT constructs in association with the implementation of e-consultations

NPT Construct	
**Coherence**	Sense-making work to understand the possibilities of an intervention. *What are the purposes of e-consultations?*
**Cognitive participation**	Relational work that builds a community of practice around an intervention. *What promotes participation with e-consultations?*
**Collective action**	Operational work that people enact to make an intervention function. *How do participants interact with e-consultations to make them work?*
**Reflexive monitoring**	Appraisal work where people assess how a new practice affects them and others. *How do participants appraise e-consultations?*

Patients' perspectives of implementing technology have been researched less;[16] and NPT may need to be developed to account for patients' implementation roles.[17-20] With e-consultations patients input details of their symptoms, which produces the e-consultation that the practice then processes. In this way, an e-consultation is co-produced; both patients and staff are integral to the process. This article examines 'co-production in the implementation of core services' where 'citizens are actively engaged in the implementation, but not the design, of an individual service'[21] (p.433). We develop NPT to analyse patients' implementation roles, using service co-production theory[21-28] to understand how both patients and staff co-implement and use technology.

We undertook an evaluation of eConsult to analyse patient usage, acceptability, effectiveness and costs of implementing the system in the 36 general practices, incorporating a quantitative, qualitative and economic analysis. The quantitative and economic analysis on usage and costs[29] and interviews with practice staff about e-consultations[30] are reported separately. This article analyses the implementation and acceptability of the eConsult system, using normalisation process theory (NPT)[13] and service co-production theory[21-28] to understand staff and patients' experiences of the system.

For peer review only - http://bmjopen.bmj.com/site/about/guidelines.xhtml

METHODS

Research design

Data was collected that covered up to 15 months usage of the system by GP practices, and consisted of three components:

1. Qualitative interviews with staff from a sample of 6 GP practices
2. Electronic medical records for patients who had conducted an e-consultation from a sample of 8 GP practices
3. Patient survey data from patients who had conducted an e-consultation about their experiences of e-consultations from all 36 GP practices.

Sampling and recruitment

To conduct qualitative staff interviews and collect anonymised patient record data, GP practices were purposively sampled to ensure a range of: locations (rural/suburban/urban); practice levels of deprivation measured by the Index of Multiple Deprivation (IMD)[31] from practice postcodes; and volume of e-consultation usage (calculated by dividing the number of e-consultations received by the number of days the system was live at time of sampling). Table 2 illustrates the range of practices recruited, with details of the eight practices purposively sampled to collect anonymised patient medical record data from e-consultations, and the six practices purposively sampled to conduct qualitative interviews. A purposive sample of staff with different professional roles from these six practices involved in the processing or managing of e-consultations were invited to be interviewed via email with participant information sheets, with contacts and invitations facilitated by practice managers. Practice protocols on using the system were also given to the researchers where they were available.

Table 2: Sampled GP practice and interview participant profiles

GP Practice	E-consultations per day live (range 0.1-2.9 for 36 practices)	IMD levels of deprivation (mean English score = 23.7 [31], high score=most deprived)	Area	% ethnic minority population	Staff interviews	Number of e-consultations randomly sampled from electronic patient record data
**1**	2.9	21.1	Urban	18.6%	2 GPs, 1 AD, 1 PM	64
**2**	0.9	8.0	Rural	1.9%	2 GPs, 2 AD, 1 PM	60
**3**	1.6	40.7	Urban	36.3%	2 GPs, 1 AD,	70

For peer review only - http://bmjopen.bmj.com/site/about/guidelines.xhtml

					1 PM	
**4**	0.2	46.7	Urban	9.4%	1 GP, 1 AD, 1 PM	0
**5**	0.7	31.3	Urban	6.1%	1 GP, 1 PM	38
**6**	0.8	13.0	Urban	11.6%	2 GPs, 1 NP, 1 AD, 1 PM	0
**7**	2.2	24.4	Urban	12.9%	0	60
**8**	1.2	11.2	Suburban	8.3%	0	60
**9**	0.6	8.0	Urban	7.9%	0	66
**10**	1.5	11.3	Urban	10.0%	0	67

For peer review only

Qualitative interviews and analysis

Interviews were conducted with 23 practice staff including: 10 GPs, 1 nurse practitioner (NP) (this interviewee is designated with (GP) notation to avoid potential identification), 6 practice managers (PM), and 6 'administrators' including an IT manager and receptionists (AD) (see Table 2). Interviews took place both face to face within general practice private offices (n=20) and over the phone (n=3), and lasted between 10-40 minutes. All participants gave full informed consent. Interviews were semi-structured, using a topic guide that had been developed with reference to NPT[13] covering: (i) introduction of e-consultations into the practice; views, promotion, training needed, (ii) e-consultation processing, (iii) impact on workload, (iv) impact on clinical practice, and (v) attitudes to future implementation. All interviews were audio recorded, transcribed, anonymised, checked for accuracy and imported into NVivo 10 software to aid analysis. Inductive thematic analysis was used grounded in the data,[32] NPT was then used as a framework to order the codes. Analysis was conducted by two researchers (MF and JB) with a subset double coded to ensure rigour. Data collection and analysis was conducted in parallel, with participants sampled until data saturation was reached. Key analytic themes were discussed within the research team to enhance credibility and external validity.

Patient record data

Electronic anonymised patient record data were collected from a random sample of patients (n=485) who had used e-consultations from eight of the participating practices (Table 2), between April 2015 to June 2016. A staff member from a participating practice retrospectively extracted anonymised patient data from patient records onto an Excel database from all practices, including: patient demographics; reason for contact; the actions taken resulting from the e-consultation (e.g. telephone call, face-to-face appointment, email advice); and further care provided by the practice in the next 30 days in relation to the e-consultation (e.g. treatment room tests, nurse appointments,

For peer review only - http://bmjopen.bmj.com/site/about/guidelines.xhtml

further GP consultations etc.). When analysing practice responses to e-consultations, the primary response was designated as the most resource-intensive action (i.e. a face to face appointment is more resource intensive than a phone appointment than a prescription), and the secondary action was the next most resource intensive (i.e. a prescription or advice) to account for multiple e-consultation processes.[29] Descriptive statistics were used to analyse these, and where appropriate, chi-square tests or odds ratios with 95% confidence intervals and p-values calculated.[29] The primary clinical reason for patients using an e-consultation was cross tabulated with the primary response to the e-consultation from practice staff (Supplementary file, Table A).

Patient survey data

Patient survey data was routinely collated by the e-consultation software company [Hurley], using their own survey design. Patients who opted in were sent a questionnaire seven days after the submission of their e-consultation. This contained both tick box questions and free text. We were given access to this anonymised data from the software company for the 36 pilot GP practices from April 2015 to June 2016. The tick box questions were analysed using descriptive statistics (Supplementary file, Table B). Free text comments were coded using inductive thematic analysis grounded in the data,[32] NPT was then used as a framework to order the codes. Answers were analysed by two researchers (MF and JB), with a subset double coded. Patient survey quotes are labelled P01, P02… in the following data analysis. From 7,472 e-consultations, a total of 751 patients (10%) submitted a survey with quantitative data, and additional comments to individual questions ranged from 38 to 512 patients (Supplementary file, Tables B-C).

Using NPT and co-production to integrate patient and staff perspectives

The NPT concepts of *coherence* and *cognitive participation* were analysed using staff interviews and patients' survey responses. *Coherence* explored staff and patients' expectations of the system and how the system's purpose and possibilities were understood. *Cognitive participation* explored the relational work that promoted engagement with e-consultations. *Collective action* explores how the system was operationalised. Process mapping using both patient and staff data enabled an understanding of how patients and staff co-implement and co-produce an e-consultation process through their interactions.[25] Initially an e-consultation workflow process map for each practice was developed from staff interviews and practice protocols on using the system. These were integrated to illustrate core practice processes. 'Touchpoints'[24 33] were identified, where patients and staff interacted through the e-consultation process.[34] Touchpoints are where 'people and tangible things' shape service experiences.[22 33-35] They have been used in co-production literature[24 33] and health service improvement methods such as experience based co-design.[36-38] Here they were used as an

For peer review only - http://bmjopen.bmj.com/site/about/guidelines.xhtml

analytic lens to examine the operational work and experiences of both staff and patients through an e-consultation. The process map and key touchpoints are illustrated in Figure 1 and analysed in the *Collective action* results section. *Reflexive monitoring* explored staff and patient appraisal of the system, analysing when e-consultations may work for whom. The cross tabulation of patients' clinical reasons for using an e-consultation with practice staff responses (Supplementary file, Table A), was combined with the analysis of staff and patients' satisfaction with the system, integrating all data sets.

RESULTS

The results are presented using the four NPT concepts, as detailed above.

Coherence

Coherence describes patients' and staff understandings of the system's purpose. E-consultations were seen by practice staff as a new and different way of working that had the potential to reduce GP workload pressures:

> *We are massively overstretched … So, that was one of the reasons why I wanted [eConsult], was so that we could make it easier… to deal with queries and often relatively simple queries that come through* (PM23).

Practices were aware of the difficulties patients faced in securing GP appointments and e-consultations were seen to provide a different pathway to care and advice. The pilot provided practice managers with an opportunity to test out the system without financial investment.

Patients saw e-consultations as a new, alternative way to communicate with their practice, that could be used out of surgery hours, from *'my bed'* (P507) or on holiday: *'It is quick and easy to use at a time to suit myself. Saves having to call the surgery'* (P61). Several patients' comments exhibited an understanding of the pressures that practices were under: '*It saves the GP time, saves me coming to the practice, great all round'* (P81).

Cognitive participation

Cognitive participation describes the relational work that people were involved in to promote participation with the system. Implementing e-consultations within practices was reported by practice staff as a relatively straight forward process, with little training needed. However, there were varying feelings toward it:

> *We were quite happy to do it* (AD08).

For peer review only - http://bmjopen.bmj.com/site/about/guidelines.xhtml

I was feeling very anxious about the extra workload … some things feel like a bottomless pit (GP22).

Practices employed different promotion methods to patients to varying degrees including through their website, waiting room banners, leaflets, social media, and newsletters. In some practices, there was a feeling that there was not as much uptake of the system as expected.

Some patients were activated to use the system because they couldn't get an appointment: *'No available appointment for 2 weeks'* (P10); *'Tried Switchboard nine times … Went online'* (P05). Others favoured the online format and remote consultation style, they used the system as it was difficult to visit the practice due to disabilities, illness or working commitments, or saw it was a more legitimate way to access GP advice: *'didn't want to waste Drs time with a full consultation which I didn't need'* (P171).

Collective action

Collective action describes how the system was operationalised in practice by patients and staff. Figure 1 maps where staff and patients interact through an e-consultation process, identifying three touchpoints[2], key interactions and experiences through the co-production of an e-consultation.

Figure 1: E-consultation process map highlighting key touchpoints

Touchpoint 1: Patient interaction with e-consultation system

Touchpoint 1 in Figure 1 relates to patients' initial decision to complete an e-consultation form, and their interaction with the system. Most patient survey respondents agreed that the system was easy to use (Supplementary file, Table B): *'had no problems at all'* (P398). It was '*helpful to be able to contact about minor requests*' (P475). Some patients preferred the written interface over a verbal conversation: '*Allowed me time to describe symptoms in greater detail than talking'* (P279). Patients reported that the system did not seem to account for multiple conditions.

Touchpoint 2: GP Practice processing of e-consultations

During the pilot, the system was not integrated with the patient record IT system [EMIS] that practices used. Administrators downloaded patients' e-consultations from the system and printed them or manually imported them into patients' records. Some administrators spoke of conducting some triage e.g. directing hay fever queries to pharmacy. Clinicians described variability in the quality of information from the e-consultation forms. Whilst information could be: '*clear and concise'* (GP13), this was not always the case:

For peer review only - http://bmjopen.bmj.com/site/about/guidelines.xhtml

One patient needed to be admitted [to hospital] ... Because the symptoms weren't very clear (GP05).

Most GPs often reverted to face to face or phone conversations to gain more information to conduct clinical decision-making.[29 30] One clinician who had substantial experience of conducting phone triage, reported that they dealt with most e-consultations without needing to see patients face-to-face, unless it was for new acute symptoms/ diagnosis. This potentially suggests that GPs may get more used to this approach over time.

Touchpoint 3: GP Practice interaction with patients following their e-consultation

Practices organised follow-up appointments in different ways. In some, a face to face appointment might have to *'start from scratch'* (GP05), because a different clinician originally dealt with the e-consultation:

I had to repeat everything I entered on line. What's the point in asking if you're not going to read it? (P90)

Other practices had more continuity where GPs could follow through the e-consultation, which provided benefits to the consultation:

The actual face to face consultation is then quicker, and that's quite nice in some ways …it doesn't open up other avenues, to a degree, okay, so it's more efficient (GP18)

Reception staff usually contacted patients via a practice email address or phone, to relay a message from a GP to patients, or to arrange the next step or outcome of their e-consultation. Occasionally, patients who had had no opportunity to speak to a doctor, were unhappy about this: '*I had no opportunity to ask any questions'* (P44). Some patients reported missing practice phone calls, one spoke of '*telephone answer machine ping-pong'* (P275). 14% of survey respondents reported not being contacted at all following their e-consultation (surveys were sent seven days after an e-consultation submission) which left patients dissatisfied (Supplementary file, Table B):

I feel like my treatment has been compromised and delayed as a result of this service (P48)

The system had an in-built function to electronically respond to a patient's email address; however only one out of six practices where interviews were conducted said they used this, and not all staff could access the system.

For peer review only - http://bmjopen.bmj.com/site/about/guidelines.xhtml

Patient record data illustrates that 38% of e-consultations resulted in a primary response of a face to face consultation.[29] Several patients commented that they had received easier access to a face to face appointment through the system:

> *The service recommended immediate attention that resulted in a quicker appointment than otherwise would have been the case* (P313)

Whilst a face to face consultation often satisfied patients, it could potentially duplicate GP workload,[30] with initial e-consultation processing by administrators and a GP, plus an appointment space. 32% of e-consultations resulted in a primary response of a phone consultation. Where e-consultations resulted in a primary response of a prescription (7.2%), a 'fit note' statement of fitness for work (3.1%), test or treatment request (1.6%), referral (1.6%) or advice (9.1%) (occurring in 23% of patient e-consultation records),[29] these could save GP time as administrative staff relayed messages and there was no direct contact between the patient and GP.

Reflexive monitoring: Who do e-consultations work for, and when?

Reflexive monitoring describes how patients and staff appraised the system, integrating all data sets to explore when e-consultations were likely to work best for whom.

Patients' satisfaction with the system was high and most (81%) were likely to recommend the system to others. 76% said they would use the service again instead of booking a face to face appointment (Supplementary file, Table B). Dissatisfaction with the system was usually a result of: lack of interaction with a GP; missed communications; thinking that their query could be answered remotely, and then being asked to book an appointment; or lack of timely follow-up of their e-consultation. Several patients suggested improvements (at touchpoint 1) that have since been integrated into the system by the software developers e.g. the ability to upload photos; being able to nominate a preferred GP; and an administration channel for requests such as a fit note or test results.

Interviews revealed that clinical staff were less satisfied with the system, as time saved in completing e-consultations without further GP-patient communication (23%), was counterbalanced by e-consultation processing and GPs needing to phone or see patients in 70% of e-consultations, which could duplicate GP work.[30] Analysing why patients were e-consulting and the resulting action (Supplementary file, Table A, and [29]), and combining this with staff and patients' appraisal of the system, Table 3 summarises when e-consultations were likely to work and be effective for patients and GPs. For GPs, it was only for relatively straightforward queries that the system could save substantial time. Patients were satisfied more often as e-consultations could: save them time, get

For peer review only - http://bmjopen.bmj.com/site/about/guidelines.xhtml

them a quicker appointment, provide an easier access route to GP services, or they preferred the remote access format.

Table 3: Nature of e-consultations and the resulting possible satisfaction and dissatisfaction of staff and patients

Nature of query	Patients' satisfaction	Practice staff satisfaction
Administrative queries	✓ Most processed remotely	✓ Most processed remotely
Medication queries and simple queries about pre-existing patient conditions	✓ Most processed remotely by phone or prescription	✓ Most processed remotely by phone or prescription
Queries about new conditions	✓ May get quicker response	X Face to face appointment more likely - possible work duplication
Complex questions, multiple symptoms	✓ May get quicker response	X Face to face appointment more likely - possible work duplication

Practice suggestions for system improvement (at touchpoint 1) included that patients could be signposted away from consulting a GP more often, to encourage more self-help or use of pharmacy when '*appropriate*' (AD11), to '*make people aware that they're in some cases wasting GP's time*' (AD04). Some practice staff suggested that patients might be guided to use e-consultations under certain conditions where only remote GP input was likely to be needed (as in Table 3). In contrast, if a patient had multiple symptoms for a new condition for which a face to face appointment was likely to be needed, GPs suggested that a modified system could flag this, directing patients not submit an e-consultation but to directly book a face to face appointment, to avoid GP work duplication.

Comparatively analysing different practice processing of e-consultations (touchpoint 2) suggests that administrative allocation of e-consultations to GPs could affect process efficiency. If administrators allocated e-consultations to a GP who had previously seen the patient (especially about similar symptoms/ conditions) this may support more efficient processing, as GPs would be more familiar with the patient and condition:

> *We like to look at each patient's notes to find out which doctor perhaps has seen this patient for that particular problem and then we would know where to direct that e-consultation* (AD09)

For peer review only - http://bmjopen.bmj.com/site/about/guidelines.xhtml

Administrators could also book face to face or phone consultations with the GP who had processed the e-consultation and was familiar with the patient query. This could focus the appointment, and avoid situations where patients felt that GPs appeared not to have read their e-consultation.

Improvements at touchpoint 3 (practice interaction with patients about e-consultations) include more robust practice communication mechanisms to reduce patient dissatisfaction about practice communication relating to their e-consultation. This could be supported by integration with electronic practice IT systems,[16] and further use of electronic communications back to patients that more staff can access and use.

Summarising this touchpoint analysis, highlights potential improvements to the system and its implementation (Table 4).

Table 4: Suggested improvements to implement the e-consultation system

**Issues identified with Touchpoint 1: Patient decides to fill in an e-consultation form**	**Suggested technological improvements**
Patients suggested several ways to improve system usability, such as: the ability to upload photos; being able to nominate a preferred GP; and an administration channel for requests such as a fit note or test results	Software developers have implemented these improvements to the system in its ongoing development
Practice staff suggested that the system could encourage more use of pharmacy or self-help options where appropriate	Better signposting to pharmacy and self-help options on website interface
Promoting patients to use e-consultations for simple conditions and questions to save face to face appointments	Appropriate patient signposting on when to complete an e-consultation
Reducing patient e-consultation usage when they need a diagnosis about new, complex and multiple symptoms	Appropriate patient signposting on when not to complete an e-consultation but to directly book a face to face appointment to save practice staff work duplication
Reducing the use of the e-consultation system to directly access face-to-face appointments	Signposting to discourage patient use of the system if they want a face to face appointment
**Issues identified with Touchpoint 2: Practice processing of e-consultations**	**Suggested practice implementation improvements**
Some GPs received e-consultations that could have been dealt with by a pharmacy	Administrative triage where appropriate e.g. directing hay fever queries to pharmacy, to save GP time
Supporting more efficient processing of e-consultations, and potentially reduce follow-on face to face consultations	Allocate e-consultations to GPs who are familiar with the patient and their symptoms, where appropriate
**Issues identified with Touchpoint 3: Practice**	**Suggested improvements**

For peer review only - http://bmjopen.bmj.com/site/about/guidelines.xhtml

interaction with patients about e-consultations	
Patient complaints that they had to repeat information in consultation as GPs appeared not to have read e-consultations	Allocate follow-on phone and face-to-face appointments to GPs who initially process e-consultations
Patients missing or not receiving communication back from the practice about their e-consultation	More robust internal practice systems to ensure that patients receive communication back about their e-consultations<br>Stronger e-consultation and practice IT integration to support electronic communications back to patients that more practice staff can access and use

None of the 36 practices took up the system after the pilot, which would have involved paying market prices for the software. However, 13 practices were interested in continuing to use the system if costs were paid for by alternative funding sources, and technological interoperability with electronic patient record systems was further developed.

DISCUSSION

Key findings

Practices were originally interested in the system to improve access and create efficiencies. Whilst some patients used the system to try and save time for both themselves and their GPs, other patients were activated to use e-consultations when they could not get a timely appointment. Because practices were dependent upon patients deciding how and when to use e-consultations, clearer guidance may be needed for patients to support more efficient use of e-consultations (see Table 4, touchpoint 1).

Our findings highlight the difficulties in substituting real time interaction with an asynchronous technological interface (touchpoints 2 and 3). This could reduce professionals' ability to use tacit knowledge of patients concerns, patients' ability to negotiate treatment options and shared decision-making. GPs often needed further information when processing e-consultations, leading to face to face and phone consultations. However, the system was being piloted, which meant that GPs were developing their skills in e-consultations, so phone and face to face consultations may decrease over time. GPs speculative fears about the perceived risks to patients of online consultations and the potential increases in workload[2 10] are to some extent causally linked through this study. The asynchronous communication mechanisms meant that GPs had to initiate face to face and phone consultations where they needed further information, which could duplicate workload. For more

For peer review only - http://bmjopen.bmj.com/site/about/guidelines.xhtml

efficient implementation of e-consultations, further consideration may be needed of when it is appropriate to use technology e.g. for less complex tasks,[9] and when face to face interaction is essential, such as in the diagnosis of complex symptoms[39] (Table 3 and 4).

Strengths and limitations

This study is one of the largest UK pilot independent evaluations of e-consultation systems within GP practices to date. The broad sample of practice staff interviewed, combined with patient survey data allows a comprehensive insight into the e-consultation system. Combining NPT and co-production theory has enabled the integration of staff's and patients' perspectives; touchpoint analysis has suggested improvements to be developed. However, because the study was based on a pilot period of one online consultation system; the issues highlighted may be a result of the system studied, rather than all online consultation systems. Patient surveys were only sent to patients who had submitted an e-consultation; thus representing a self-selecting sample of those who had invested time into the system. Because usage was low,[29] those patients using the system may be unrepresentative of the wider patient population.

Policy and practice implications

Technology is often promoted to improve NHS efficiency,[40] but benefits are often more limited due to implementation difficulties.[16] In this study, no practices experienced sufficient workload savings to warrant practices own financial investment in the system at current market prices, however the system did improve access for some patient groups. NHS England has offered financial support for practices to adopt online consultations, this research affirms that clear implementation guidance is also needed.[41] This study has suggested improvements to support future implementation and technological development of e-consultation systems (Table 4).

CONCLUSIONS

E-consultations can increase patient access and satisfaction, but in their current form, were not perceived as creating sufficient workload efficiencies for continued practice usage. Patients' use of e-consultations impacted upon staff's appraisal of the system. Where both patients and staff interact with healthcare technology, it is in effect 'co-implemented'. Mapping the co-production of an e-consultation through touchpoints[33 34] has highlighted where the system may be redesigned or implementation improved. This analysis can support more effective implementation of appropriate technology that accounts for professional and patient experiences.

For peer review only - http://bmjopen.bmj.com/site/about/guidelines.xhtml

FOOTNOTES

Acknowledgements

Authors thank staff from participating practices for assisting with the collection of individual-level data, and the software developers for providing web usage statistics and anonymous patient survey data.

Funding

This research is funded by the One Care Consortium Ltd and the National Institute for Health Research (NIHR) Collaboration for Leadership in Applied Health Research and Care West at University Hospitals Bristol NHS Foundation Trust. The One Care Consortium facilitated data collection. The views expressed are those of the authors and not necessarily those of the NHS, the NIHR or the Department of Health.

Competing interests

None declared.

Ethical approval

The study was reviewed by the NHS Health Research Authority (project ID: 204925) and ethically reviewed by the University of Bristol, Faculty of Health Sciences, Research Ethics Committee (Application 32961).

Contributors

KN, JH, HE, MF, JB, and EB were responsible for the study design and collection of data. KN, JH and EB were responsible for study management and co-ordination. CS was a project advisor throughout. MF, JB, HE and KN analysed the data. MF drafted the paper. All authors read, commented on and approved the final manuscript.

Data sharing statement

The datasets analysed during the current study are not publicly available, as participants were not asked to consent to this at the time of data collection. Related patient survey results are available in the Supplementary file.

Supplementary file

Table A: Reason for e-consultation by primary response from practice staff (patient record data)

Table B: Patient survey – how and why accessed e-consultations, and resulting satisfaction

Table C: E-consultation patient survey free text response numbers

For peer review only

REFERENCES

1. Hobbs FDR, Bankhead C, Mukhtar T, et al. Clinical workload in UK primary care: a retrospective analysis of 100 million consultations in England, 2007-2014. *Lancet* 2016;387(10035):2323-30. doi: 10.1016/S0140-6736(16)00620-6
2. BMA. National survey of GPs: the future of general practice. London: British Medical Association 2015.
3. Kaffash J. Average waiting time for GP appointment increases 30% in a year 2016 [updated 10 June 2016]. http://www.pulsetoday.co.uk/your-practice/access/average-waiting-time-for-gp-appointment-increases-30-in-a-year/20032025.article.
4. Atherton H, Ziebland S. What do we need to consider when planning, implementing and researching the use of alternatives to face-to-face consultations in primary healthcare? *Digit Health* 2016;2:2055207616675559. doi:10.1177/2055207616675559
5. NHSEngland. GP Access Fund: NHS England; 2013. https://www.england.nhs.uk/gp/gpfv/redesign/improving-access/gp-access-fund/ (accessed 17 Jan 2017).
6. NHSEngland. General Practice Forward View. NHS England, 2016.
7. NHSEngland. Online Consultation Systems: NHS England; 2016. https://www.england.nhs.uk/gp/gpfv/redesign/gpdp/consultation-systems/ (accessed 10 Feb 2017).
8. Hanna L, May C, Fairhurst K. Non-face-to-face consultations and communications in primary care: the role and perspective of general practice managers in Scotland. *Inform Prim Care* 2011;19(1):17-24.
9. Hanna L, May C, Fairhurst K. The place of information and communication technology-mediated consultations in primary care: GPs' perspectives. *Fam Pract* 2012;29(3):361-6. doi: 10.1093/fampra/cmr087
10. Brant H, Atherton H, Ziebland S, et al. Using alternatives to face-to-face consultations: a survey of prevalence and attitudes in general practice. *Br J Gen Pract* 2016 doi: 10.3399/bjgp16X685597
11. The One Care Consortium. http://onecareconsortium.co.uk/about/pmcf-programme/ (accessed 17 Jan 2017).
12. EMISHealth. eConsult: A platform that enables patients to self-manage and consult online: Emis Health; 2016. https://www.emishealth.com/products/econsult/ (accessed 30 Jun 2016).
13. May C, Finch T. Implementing, Embedding, and Integrating Practices: An Outline of Normalization Process Theory. *Sociology* 2009;43(3):535-54. doi: 10.1177/0038038509103208
14. Murray E, Treweek S, Pope C, et al. Normalisation process theory: a framework for developing, evaluating and implementing complex interventions. *BMC Med* 2010;8(1):1-11. doi: 10.1186/1741-7015-8-63
15. May C, Rapley T, Mair FS, et al. Normalization Process Theory On-line Users' Manual, Toolkit and NoMAD instrument. 2015. http://www.normalizationprocess.org.
16. Ross J, Stevenson F, Lau R, et al. Factors that influence the implementation of e-health: a systematic review of systematic reviews (an update). *Implement Sci* 2016;11(1):146. doi: 10.1186/s13012-016-0510-7
17. Gibson J, Lightbody E, McLoughlin A, et al. 'It was like he was in the room with us': patients' and carers' perspectives of telemedicine in acute stroke. *Health Expect* 2016;19(1):98-111. doi: 10.1111/hex.12333
18. Kennedy A, Vassilev I, James E, et al. Implementing a social network intervention designed to enhance and diversify support for people with long-term conditions. A qualitative study. *Implement Sci* 2016;11(1):1-15. doi: 10.1186/s13012-016-0384-8

For peer review only - http://bmjopen.bmj.com/site/about/guidelines.xhtml

19. Clarke DJ, Godfrey M, Hawkins R, et al. Implementing a training intervention to support caregivers after stroke: a process evaluation examining the initiation and embedding of programme change. *Implement Sci* 2013;8(1):96. doi: 10.1186/1748-5908-8-96
20. Connell LA, McMahon NE, Harris JE, et al. A formative evaluation of the implementation of an upper limb stroke rehabilitation intervention in clinical practice: a qualitative interview study. *Implement Sci* 2014;9(1):1-12. doi: 10.1186/s13012-014-0090-3
21. Brandsen T, Honingh M. Distinguishing Different Types of Coproduction: A Conceptual Analysis Based on the Classical Definitions. *Publ Admin Rev* 2016;76(3):427-35. doi: 10.1111/puar.12465
22. Osborne SP, Radnor Z, Strokosch K. Co-Production and the Co-Creation of Value in Public Services: A suitable case for treatment? *Public Manag Rev* 2016;18(5):639-53. doi: 10.1080/14719037.2015.1111927
23. Batalden M, Batalden P, Margolis P, et al. Coproduction of healthcare service. *BMJ Qual Saf* 2015 doi: 10.1136/bmjqs-2015-004315
24. Radnor Z, Osborne SP, Kinder T, et al. Operationalizing Co-Production in Public Services Delivery: The contribution of service blueprinting. *Public Manag Rev* 2014;16(3):402-23. doi: 10.1080/14719037.2013.848923
25. Alford J. Co-Production, Interdependence and Publicness: Extending public service-dominant logic. *Public Manag Rev* 2016;18(5):673-91. doi: 10.1080/14719037.2015.1111659
26. Ordanini A, Pasini P. Service co-production and value co-creation: The case for a service-oriented architecture (SOA). *EMJ* 2008;26(5):289-97. doi: 10.1016/j.emj.2008.04.005
27. Osborne SP, Radnor Z, Nasi G. A New Theory for Public Service Management? Toward a (Public) Service-Dominant Approach. *ARPA* 2013;43(2):135-58. doi: 10.1177/0275074012466935
28. Osborne SP, Strokosch K. It takes Two to Tango? Understanding the Co-production of Public Services by Integrating the Services Management and Public Administration Perspectives. *British Journal of Management* 2013;24:S31-S47. doi: 10.1111/1467-8551.12010
29. Edwards H, Marques E, Hollingworth W, et al. Use of a primary care online consultation system, by whom, when and why: evaluation of a pilot observational study in 36 general practices in South West England. *BMJ Open* 2017;In Press
30. Banks J, Farr M, Salisbury C, et al. The use of an electronic consultation system in primary care: views and experiences from general practice. *Br J Gen Pract* 2017;In press
31. DCLG. English indices of deprivation: Department of Communities and Local Government; 2015. https://www.gov.uk/government/collections/english-indices-of-deprivation (accessed 15 Jan 2016).
32. Braun V, Clarke V. Using thematic analysis in psychology. *Qualitative Research in Psychology* 2006;3:77-101.
33. Parker S, Heapy J. The Journey to the Interface: How public service design can connect users to reform. London: Demos, 2006.
34. Bitner MJ, Ostrom AL, Morgan FN. Service Blueprinting: A practical technique for service innovation. *Calif Manage Rev* 2008;50(3):66-94.
35. Normann R. Service Management: Strategy and Leadership in Service Business. Third ed. Chicester: John Wiley and Sons 2000.
36. Bate P, Robert G. Bringing user experience to healthcare improvement: the concepts, methods and practices of experience-based design. Oxford: Radcliffe Publishing 2007.
37. Boaz A, Robert G, Locock L, et al. What patients do and their impact on implementation. *J Health Organ Manag* 2016;30(2):258-78. doi: 10.1108/jhom-02-2015-0027
38. Wherton J, Sugarhood P, Procter R, et al. Co-production in practice: how people with assisted living needs can help design and evolve technologies and services. *Implement Sci* 2015;10:75. doi: 10.1186/s13012-015-0271-8
39. Jiwa M, Meng X. Video Consultation Use by Australian General Practitioners: Video Vignette Study. *J Med Internet Res* 2013;15(6):e117. doi: 10.2196/jmir.2638

For peer review only - http://bmjopen.bmj.com/site/about/guidelines.xhtml

40. Greenhalgh T, Procter R, Wherton J, et al. The organising vision for telehealth and telecare: discourse analysis. *BMJ Open* 2012;2(4) doi: 10.1136/bmjopen-2012-001574
41. Stokes-Lampard H, Stodel M, Bullard E, et al. GP Forward View: Assessment of progress Year 1. London: Royal College of General Practitioners, 2017.

Figure 1: E-consultation process map highlighting key touchpoints

For peer review only

For peer review only - http://bmjopen.bmj.com/site/about/guidelines.xhtml

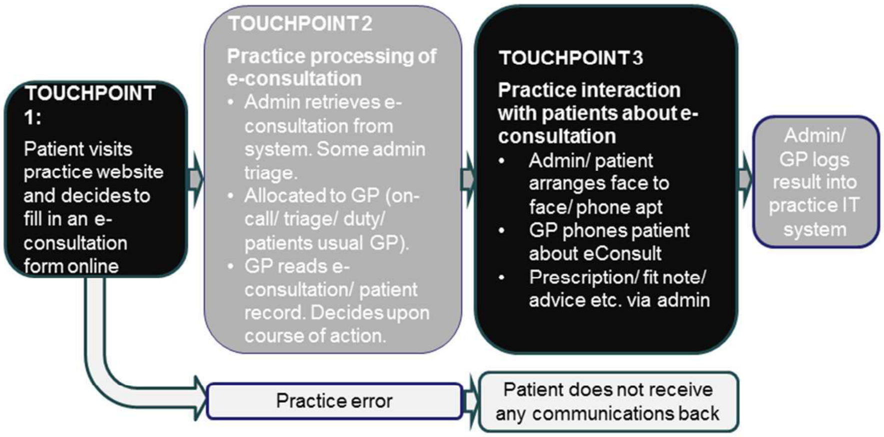

Figure 1: E-consultation process map highlighting key touchpoints

254x190mm (96 x 96 DPI)

SUPPLEMENTARY FILE

Table A: Reason for e-consultation by primary response from practice staff (patient record data)

Table A (Additional file) cross tabulates the primary clinical reason for patients using an e-consultation with the primary response to the e-consultation from practice staff. When analysing practice responses to e-consultations, the primary response was defined as the most resource-intensive action (i.e. a face to face appointment is more resource intensive than a phone appointment than a prescription). This ensured that where there were multiple e-consultation processes, the most resource intense of these was accounted for.

Reason	Total number	Face to face	Phone consult	Prescription	Fit note	Test request	Refer routine	advice	Other/unknown
Musculoskeletal / limb pain	60	48.3%	38.3%	1.7%	0	1.7%	3.3%	1.7%	0
Infection/Immunological	70	40.0%	41.4%	8.6%	0	0	0	0	0
Neurological	26	53.9%	26.9%	0	0	3.9%	0	0	3.9%
Sexual/Reproductive health	41	39.0%	41.5%	7.3%	0	4.9.%	0	0	2.4%
Dermatological	33	48.5%	21.2%	18.2%	0	0	0	3.0%	0
Respiratory	25	52.0%	24.0%	4.0%	0	0	0	0	8.0%
Mental health	29	44.8%	34.5%	10.3%	0	0	0	0	0
Digestive	19	52.6%	26.3%	5.3%	0	0	0	0	5.3%
Medication query/advice	19	0	73.7%	10.5%	0	0	0	0	5.3%
Administrative [a]	107	12.2%	27.1%	11.2%	14.0%	1.9%	5.6%	10.3%	7.5%
Other / Unclear	53	38.4%	17.0%	0	0	3.8%	0	5.7%	1.9%

[a] fit notes, test results, referrals, repeat scripts, letter requests, booking appointments

Table B: Patient survey – how and why accessed e-consultations, and resulting satisfaction

Some of these questions are expanded further with free-text responses, overviewed in Table C.

	% (n)
How did you hear about the Consult Online from Home Service?	
My GP told me about it	0.3% (2)
Someone else from the GP practice	4.2% (33)
From the GP practice website	58.0% (455)
Another patient/family member	4.2% (33)
From an internet search	5.6% (44)
I read about it	2.7% (21)
From a leaflet or promotional banner	18.5% (145)
Other → Free text	6.6% (52)

For peer review only

	% (n)
What was the medical reason for using the Consult Online From Home Service?	
To request a new prescription	11.1% (88)
To request a repeat prescription	0.5% (4)
To request my GP's feedback about an ongoing condition	29.4%(239)
To request my GP's feedback about a new condition	41.5% (329)
To request administrative help, such as a sick note or referral letter	9.3% (74)
Other → Free text	8.2% (65)
Was there a practical reason why you used the Consult Online from Home Service?	
I didn't have time to come in	13.2% (81)
I wasn't able to have the appointment time I wanted	9.3% (57)
I wanted to try out the service as an alternative to an appointment	69.0% (422)
No practical reason	5.4% (33)
Other → Free text	3.1% (119)
When your practice contacted you about your Consult Online From Home Service assessment, what were you advised to do?	
How to look after the problem myself, without contacting the GP practice/other health service	9.1% (56)
Pick up a prescription from the surgery /pharmacy	27.6% (169)
Visit the GP practice for face-to-face at later date	40.0% (244)
Go to A&E department / Walk-in Centre etc	1.5% (9)
Was not contacted	13.7% (84)
Other → Free text	8.3% (51)
How likely are you to recommend the Consult Online From Home Service to friends and family if they need similar care or advice?	
Extremely likely	55.5% (422)
Likely	25.2% (192)
Neither likely nor unlikely	7.2% (55)
Unlikely	6.6% (50)
Extremely unlikely	5.5% (42)
Would you use the service again instead of booking a face to face appointment?	
Yes	76.3% (582)
No	8.5% (65)
Not sure	15.2% (116)
Thinking about your use of the Consult Online From Home Service, would you agree or disagree with the statements below	
The Consult Online From Home Service was easy to use.	
Strongly agree	50.7% (383)
Agree	37.6% (284)
Neither agree nor disagree	5.2% (39)
Disagree	5.2% (39)
Strongly disagree	1.5% (11)

For peer review only

	% (n)
Overall, how satisfied or dissatisfied were you with using the Consult Online From Home Service for your health assessment?	
Very satisfied	60.3% (459)
Fairly satisfied	20.4% (155)
Neither satisfied nor dissatisfied	4.3% (33)
Fairly dissatisfied	7.4% (56)
Very dissatisfied	7.6% (58)

Table C: E-consultation patient survey free text response numbers

Table C summarises the free text questions from the eConsult online patient satisfaction survey, with response numbers.

**E-consultation survey question**	**Number of free text responses**
How did you hear about the Consult Online?	49
If the Consult Online service had not been available, what would you have done about your health problem?	37
Was there a practical reason why you used the Consult Online?	182
When your practice contacted you about your Consult Online assessment, what were you advised to do?	224
Did you follow the Consult Online advice? (if answered no, reason why they did not follow advice)	74
Did any of our staff make your experience particularly good?	343
Overall, how satisfied or dissatisfied were you with using the Consult Online Service for your health assessment? Reason for this	510
What improvements would you make to the service?	512

For peer review only

COREQ Statement

Implementing online consultations in primary care: A mixed method evaluation extending normalisation process theory through service co-production

Domain 1: Research team and reflexivity

Personal Characteristics

1. Interviewer/facilitator. Which author/s conducted the interview or focus group?

Jon Banks (JB)
Michelle Farr (MF)
(Included as authors, p.1, Title page)

2. Credentials. What were the researcher's credentials? E.g. PhD, MD.

JB: BA (WEngland), PhD (Wales), PGdip (Wales)
MF: BSc (Hons), MPhil, PhD, FHEA
(page number not applicable)

3. Occupation. What was their occupation at the time of the study?

JB: Research Fellow: Applied Social Science (Qualitative), National Institute for Health Research, Collaborations for Leadership in Applied Health Research and Care West (NIHR CLAHRC West), University of Bristol

MF: Senior Research Associate in Applied Social Science (Qualitative) Research, National Institute for Health Research, Collaborations for Leadership in Applied Health Research and Care West (NIHR CLAHRC West)
(institutional affiliations given, p.1)

4. Gender. Was the researcher male or female?

JB: Male
MF: Female.
(page number not applicable)

5. Experience and training. What experience or training did the researcher have?

JB: Post graduate diploma in social science research methods as part of PhD, experience of collecting and analysing qualitative data with 15 years as a research associate and research fellow.
MF: Extensive training in qualitative research methods (MPhil, PhD), taught qualitative research methods to undergraduates. 12 years' experience conducting qualitative research.
(page number not applicable)

Relationship with participants

6. Relationship established. Was a relationship established prior to study commencement?

No relationship was established before the commencement of study (p.5 gives details of how practices and participants were sampled).

7. Participant knowledge of the interviewer. What did the participants know about the researcher? e.g. personal goals, reasons for doing the research?

The professional goals of the researchers were to complete the aims and objectives of the study only. The researchers had no personal goals or reasons for doing the research. As part of recruitment and gaining informed consent clinicians were fully informed about the aims and objectives of the study through participant information sheets (p.5).

For peer review only

8. Interviewer characteristics. What characteristics were reported about the interviewer/facilitator? e.g. Bias, assumptions, reasons and interests in the research topic.

The interviewers (JB and MF) were both social scientists.

All participants were aware that the interviews were for independent academic research through participant information sheets (p.5).

The researchers had no personal interests in the study, it was solely their professional role.

Domain 2: study design

Theoretical framework

9. Methodological orientation and Theory. What methodological orientation was stated to underpin the study? e.g. grounded theory, discourse analysis, ethnography, phenomenology, content analysis

In the Background section we explain how we use normalisation process theory and service co-production theory to understand how both patients and staff co-implement and use technology (p.4).

In the Methods section we explain how NPT and co-production theory were used to integrate staff and patient perspectives on e-consultations (p.7 Heading: Using NPT and co-production to integrate patient and staff perspectives).

Participant selection

10. Sampling. How were participants selected? e.g. purposive, convenience, consecutive, snowball.

GP practices were purposively sampled to ensure a range of: locations (rural/suburban/urban); practice levels of deprivation measured by the Index of Multiple Deprivation (IMD) from practice postcodes; and volume of e-consultation usage (calculated by dividing the number of e-consultations received by the number of days the system was live at time of sampling). Six practices were purposively sampled to conduct qualitative interviews. A purposive sample of staff with different professional roles from these six practices involved in the processing or managing of e-consultations were invited to be interviewed via email, with contacts and invitations facilitated by practice managers. Please see Methods section Sampling and Recruitment (p.5) and Table 2.

11. Method of approach. How were participants approached? e.g. face-to-face, telephone, mail, email

Six practices were sampled to be invited to take part in the qualitative research, with six replacements with a similar profile if any of the first six did not want to participate. Practices were initially approached and invited to take part in the research by OneCare staff who were the study collaborators and, as a GP consortium, had direct contact with the practices. OneCare staff phoned practices and emailed them a letter to explain about the research and what getting involved would mean in terms of time and resources. If practices were interested in taking part and wanted to be contacted by the researchers, OneCare staff gave the researchers the practice managers email and telephone number to arrange a discussion about getting involved in the research. Once practice managers had agreed to take part in the research they identified who may be appropriate staff to be interviewed, focusing on those who were involved in the e-consultation system. Practice managers initially approached GPs and administrative staff to ask if they were interested in participating in research interviews. All interviewees were given participant information sheets either via the practice manager, and/ or by researchers. Interviews were organised with different staff via the practice manager. Before interviews commenced, researchers went through the participant information sheet with participants, with further opportunities to ask questions about the research. See Heading: Sampling and recruitment (p.5).

12. Sample size. How many participants were in the study?

We interviewed 23 practice staff. Please see Table 2: Sampled GP practice and interview participant profiles (p.5-6).

For peer review only

13. Non-participation. How many people refused to participate or dropped out? Reasons
Two practices did not want to take part, so we invited those reserve practices that had a similar profile to the ones who did not want to take part. Both two reserve practices agreed to take part. Because practice managers spoke to practice staff initially about taking part in interviews (p.5) we do not know how many practice staff declined to take part in an interview, as we only spoke to those who were willing to take part.

Setting
14. Setting of data collection. Where was the data collected? e.g. home, clinic, workplace
Interviews took place both face to face within general practice offices (n=20) and over the phone (n=3) (p.6).

15. Presence of non-participants. Was anyone else present besides the participants and researchers?
Most interviews took place in private offices, with just the interviewee. However, two of the practice manager interviews and two of the interviews with administrative staff were held in shared office spaces but as this was about work based processes we do not believe that this inhibited the interviews in any way (page number not applicable).

16. Description of sample. What are the important characteristics of the sample? e.g. demographic data, date.
Interviews were conducted with 23 practice staff including: 10 GPs, 1 nurse practitioner (NP) (this interviewee is designated with (GP) notation to avoid potential identification), 6 practice managers (PM), 6 'administrators' including an IT manager and receptionists (AD). Please see Table 2: Sampled GP practice and interview participant profiles and Heading: Qualitative interviews and analysis (p.5-6).

Data collection
17. Interview guide. Were questions, prompts, guides provided by the authors? Was it pilot tested?
A semi-structured, topic guide was used for all interviews, guided by NPT. This was discussed and agreed with OneCare staff, the research partners (Heading: Qualitative interviews and analysis, p.6).

18. Repeat interviews.
We did not conduct repeat interviews (p.5-6 gives the list of interviews and interviewees).

19. Audio/visual recording. Did the research use audio or visual recording to collect the data?
Yes audio recording was used through interview data collection, with full informed consent (Heading: Qualitative interviews and analysis, p.6).

20. Field notes. Were field notes made during and/or after the interview or focus group?
Yes, some summary notes were made in addition to the voice recorded interviews (page number not applicable).

21. Duration. What was the duration of the interviews or focus group?
Interviews lasted between 10-40 minutes (Heading: Qualitative interviews and analysis, p.6).

22. Data saturation. Was data saturation discussed?
Data saturation was discussed in research management meetings. Data collection and analysis was conducted in parallel, with participants sampled until data saturation was reached (Heading: Qualitative interviews and analysis, p.6).

For peer review only

23. Transcripts returned. Were transcripts returned to participants for comment and/or correction?
No. We did not feel this was possible to offer in the time available in the study (page number not applicable).

Domain 3: analysis and findings
Data analysis
24. Number of data coders. How many data coders coded the data?
Analysis was conducted by two researchers (MF and JB) with a subset double coded to ensure rigour. Key themes were discussed within the research team to enhance credibility and external validity (Heading: Qualitative interviews and analysis, p.6).

25. Description of the coding tree. Did authors provide a description of the coding tree?
The coding tree is not included in the manuscript due to word limits (page number not applicable).

26. Derivation of themes. Were themes identified in advance or derived from the data?
Themes were derived inductively from the data. These were then ordered using NPT as a framework (Heading: Qualitative interviews and analysis, p.6 and Heading: Using NPT and co-production to integrate patient and staff perspectives, p.7).

27. Software. What software, if applicable, was used to manage the data?
We used NVivo 10 qualitative software package to manage the data (Heading: Qualitative interviews and analysis, p.6).

28. Participant checking. Did participants provide feedback on the findings?
Participants did not provide feedback on the initial findings. However emerging research themes were discussed in research management meetings with OneCare, as research collaborators (page number not applicable).

29. Quotations presented. Were participant quotations presented to illustrate the themes / findings? Was each quotation identified? e.g. participant number
Yes unique participant codes are used alongside quotes, to illustrate findings. The professional roles of the interviewees were identified within the codes. (Heading: Results, p.8-14)

30. Data and findings consistent. Was there consistency between the data presented and the findings?
Yes (Heading: Results, p.8-14)

31. Clarity of major themes. Were major themes clearly presented in the findings?
Yes (Heading: Results, p.8-14)

32. Clarity of minor themes. Is there a description of diverse cases or discussion of minor themes?
Yes a range of practitioner perspectives are included (Heading: Results, p.8-14). For example, one clinician who had substantial experience of conducting phone triage, reported that they dealt with most e-consultations without needing to see patients face-to-face, unless it was for new acute symptoms/ diagnosis. This potentially suggests that GPs may get more used to this consultation approach over time (p.10).

For peer review only

BMJ Open

Implementing online consultations in primary care: A mixed method evaluation extending normalisation process theory through service co-production

Journal:	*BMJ Open*
Manuscript ID	bmjopen-2017-019966.R1
Article Type:	Research
Date Submitted by the Author:	12-Dec-2017
Complete List of Authors:	Farr, Michelle; University of Bristol, Population Health Sciences, Bristol Medical School; NIHR CLAHRC West, University Hospitals Bristol NHS Foundation Trust, 9th Floor, Whitefriars, Lewins Mead<br>Banks, Jonathan; University of Bristol, Population Health Sciences, Bristol Medical School; NIHR CLAHRC West, , University Hospitals Bristol NHS Foundation Trust<br>Edwards, Hannah; University of Bristol, Population Health Sciences, Bristol Medical School; NIHR CLAHRC West, University Hospitals Bristol NHS Foundation Trust<br>Northstone, Kate; University of Bristol, Population Health Sciences, Bristol Medical School; ALSPAC, University of Bristol<br>Bernard, Elly; One Care (BNSSG) Ltd<br>Salisbury, Chris; University of Bristol, Population Health Sciences, Bristol Medical School; NIHR CLAHRC West, University Hospitals Bristol NHS Foundation Trust<br>Horwood, Jeremy; University of Bristol, UK, Population Health Sciences, Bristol Medical School; NIHR CLAHRC West, University Hospitals Bristol NHS Foundation Trust
**Primary Subject Heading**:	General practice / Family practice
Secondary Subject Heading:	Health informatics
Keywords:	Co-production, Telemedicine < BIOTECHNOLOGY & BIOINFORMATICS, PRIMARY CARE, normalisation process theory, online consultations, touchpoints

SCHOLARONE™
Manuscripts

Implementing online consultations in primary care: A mixed method evaluation extending normalisation process theory through service co-production

Research article

Authors

Michelle Farr[1, 2] M.Farr@bristol.ac.uk
Jon Banks[1, 2] Jon.Banks@bristol.ac.uk
Hannah Edwards[1, 2] Hannah.Edwards@bristol.ac.uk
Kate Northstone[1, 2] Kate.Northstone@bristol.ac.uk
Elly Bernard[3] elly.bernard@onecare.org.uk
Chris Salisbury[1, 2] C.Salisbury@bristol.ac.uk
Jeremy Horwood[1, 2] J.Horwood@bristol.ac.uk

1. Population Health Sciences, Bristol Medical School, University of Bristol, Canynge Hall, 39 Whatley Road, Bristol, BS8 2PS, UK.

2. National Institute for Health Research Collaboration for Leadership in Applied Health Research and Care (NIHR CLAHRC) West at University Hospitals Bristol NHS Foundation Trust, 9th Floor, Whitefriars, Lewins Mead, Bristol, BS1 2NT

3. One Care (BNSSG) Ltd, Unit 4 Osprey Court, Hawkfield Business Park, Whitchurch, Bristol, BS14 0BB.

Corresponding Author:

Michelle Farr

NIHR CLAHRC West

9th Floor Whitefriars

Lewins Mead

Bristol BS1 2NT

M.Farr@bristol.ac.uk

0117 342 7279

Word count:

4,970

For peer review only

ABSTRACT

Objectives: To examine patient and staff views, experiences and acceptability of a UK primary care online consultation system and ask how the system and its implementation may be improved.

Design: Mixed method evaluation of a primary care e-consultation system.

Setting: Primary care practices in south-west England.

Methods: Qualitative interviews with 23 practice staff in 6 practices. Patient survey data for 756 e-consultations from 36 practices, with free text survey comments from 512 patients, were analysed thematically. Anonymised patients' records were abstracted for 485 e-consultations from 8 practices, including consultation types and outcomes. Descriptive statistics were used to analyse quantitative data. Analysis of implementation and usage of the e-consultation system was informed by: (a) normalisation process theory, (b) a framework that illustrates how e-consultations were co-produced, and (c) patients' and staff touchpoints.

Results: We found different expectations between patients and staff on how to use e-consultations 'appropriately'. Whilst some patients used the system to try and save time for themselves and their GPs, some used e-consultations when they could not get a timely face-to-face appointment. Most e-consultations resulted in either follow-on phone (32%) or face-to-face appointments (38%) and GPs felt that this duplicated their workload. Patient satisfaction of the system was high, but a minority were dissatisfied with practice communication about their e-consultation.

Conclusions: Where both patients and staff interact with technology, it is in effect 'co-implemented'. How patients used e-consultations impacted upon practice staff's experiences and appraisal of the system. Overall, the e-consultation system studied could improve access for some patients, but in its current form, it was not perceived by practices as creating sufficient efficiencies to warrant financial investment. We illustrate how this e-consultation system and its implementation can be improved, through mapping the co-production of e-consultations through touchpoints.

Keywords

Co-production, e-health, telemedicine, normalisation process theory, online consultations, primary care, touchpoints

Strengths and limitations of this study

- Largest UK study to date examining staff and patient experiences of using a primary care online consultation system.

For peer review only - http://bmjopen.bmj.com/site/about/guidelines.xhtml

- Extending normalisation process theory with service co-production theory enables an in-depth understanding of how patients and staff interacted with the e-consultation system.
- Touchpoint analysis enables improvements to be suggested to develop the design and implementation of online consultation systems, aimed at software designers, policymakers and general practices interested in this technology.
- This observational study was based on a pilot period of one online consultation system; and issues highlighted may be due to the system studied, rather than all online consultation systems.

BACKGROUND

English general practice clinical workload has risen by 16% over the period 2007-14.[1] 93% of general practitioners (GPs) say their workload has negatively impacted on quality of care given to patients.[2] Average waiting times for an appointment have been reported as just under 13 days.[3] Internationally, policymakers are advocating technological alternatives to face-to-face primary care consultations to improve service quality.[4] UK policy to improve primary care access includes the Prime Minister's Challenge Fund (now the GP Access Fund),[5] and the General Practice Forward View.[6] These promote greater use of technology to manage workload and improve patient access, with £45 million made available to support the implementation of online consultation systems.[7] Online or e-consultations enable patients to contact their GP via a mobile app or online portal.[7] General practice staff attitudes toward electronically based consultations include concerns about medico-legal issues, clinical limitations and increasing workload concerns.[8-10] Research into practitioners' and patients' actual experiences of e-consultations is limited, but timely, as implementation is at an early stage.[4 10]

A consortium of general practices in south west England [One Care],[11] received funding through the GP Access Fund,[5] to pilot online consultations. Starting in April 2015, the eConsult system[12] (previously called WebGP) was implemented free of charge into 36 general practices. The eConsult system was designed by GPs, software programmers and operational managers, with support from medical defence organisations.[13] Patients access the eConsult system (referred to as 'the system' in this paper) via their own GP practice website. They can access self-help, pharmacy advice, 111 (NHS non-emergency telephone advice), administrative help (such as repeat prescriptions), or submit an online form with details of their condition, electronically sending this to their GP practice, where it is then processed. If the system identifies signs or symptoms that may require immediate medical attention, patients are redirected to appropriate services, otherwise the system informs patients that their GP practice will contact them by the end of the next working day.

For peer review only - http://bmjopen.bmj.com/site/about/guidelines.xhtml

Normalisation process theory (NPT) illustrates issues to address when implementing a technology or complex intervention (Figure 1).[14-16]

Figure 1: NPT constructs in association with the implementation of e-consultations

Patients' perspectives of implementing technology have been researched less;[17] and NPT may need to be developed to account for patients' implementation roles.[18-21] With e-consultations patients input details of their symptoms, which produces the e-consultation that the practice then processes. In this way, an e-consultation is co-produced; both patients and staff are integral to the process. This article examines 'co-production in the implementation of core services' where 'citizens are actively engaged in the implementation, but not the design, of an individual service'[22] (p.433). We develop NPT to analyse patients' implementation roles, using service co-production theory[22-29] to understand how both patients and staff co-implement and use technology.

We undertook an evaluation of eConsult to analyse patient usage, acceptability, effectiveness and costs of implementing the system in the 36 general practices, incorporating a quantitative, qualitative and economic analysis. The quantitative and economic analysis on usage and costs[30] and interviews with practice staff about e-consultations[31] are reported separately. This article analyses the implementation and acceptability of the eConsult system from patient and staff perspectives, using normalisation process theory (NPT)[14] and service co-production theory[22-29] to understand their experiences and how the e-consultation system and its implementation may be improved.

METHODS

Research design

Data was collected that covered up to 15 months usage of the system by GP practices, and consisted of three components:

1. Qualitative interviews with staff from a sample of 6 GP practices
2. Quantitative data from electronic medical records for patients who had conducted an e-consultation from a sample of 8 GP practices
3. Quantitative and qualitative patient survey data from patients who had conducted an e-consultation about their experiences of e-consultations from all 36 GP practices.

Sampling and recruitment

To conduct qualitative staff interviews and collect anonymised patient record data, GP practices were purposively sampled to ensure a range of: locations (rural/suburban/urban); practice levels of deprivation measured by the Index of Multiple Deprivation (IMD) from practice postcodes; and

For peer review only - http://bmjopen.bmj.com/site/about/guidelines.xhtml

volume of e-consultation usage (calculated by dividing the number of e-consultations received by the number of days the system was live at time of sampling). Table 1 illustrates the range of practices recruited, with details of the eight practices purposively sampled to collect anonymised patient medical record data from e-consultations, and the six practices purposively sampled to conduct qualitative interviews. A purposive sample of staff with different professional roles from these six practices involved in the processing or managing of e-consultations were invited to be interviewed via email with participant information sheets, with contacts and invitations facilitated by practice managers. Practice protocols on using the system were also given to the researchers where they were available.

Table 1: Sampled GP practice and interview participant profiles

GP Practice	E-consultations per day live (range 0.1-2.9 for 36 practices)	IMD deciles of deprivation[32] (lower decile=more deprived)	Area	% ethnic minority population	Staff interviews	Number of e-consultations randomly sampled from electronic patient record data
**1**	2.9	5	Urban	17.5–20%	2 GPs, 1 AD, 1 PM	64
**2**	0.9	10	Rural	0–2.5%	2 GPs, 2 AD, 1 PM	60
**3**	1.6	1	Urban	35–37.5%	2 GPs, 1 AD, 1 PM	70
**4**	0.2	1	Urban	7.5-10%	1 GP, 1 AD, 1 PM	0
**5**	0.7	3	Urban	5-7.5%	1 GP, 1 PM	38
**6**	0.8	8	Urban	10-12.5%	2 GPs, 1 NP, 1 AD, 1 PM	0
**7**	2.2	5	Urban	12.5-15%	0	60
**8**	1.2	9	Suburban	7.5-10%	0	60
**9**	0.6	10	Urban	7.5-10%	0	66
**10**	1.5	9	Urban	7.5-10%	0	67

Qualitative interviews and analysis

Interviews were conducted with 23 practice staff including: 10 GPs, 1 nurse practitioner (NP) (this interviewee is designated with (GP) notation to avoid potential identification), 6 practice managers (PM), and 6 'administrators' including an IT manager and receptionists (AD) (see Table 1). Interviews took place both face to face within general practice private offices (n=20) and over the phone (n=3),

For peer review only - http://bmjopen.bmj.com/site/about/guidelines.xhtml

and lasted between 10-40 minutes. All participants gave full informed consent. Interviews were semi-structured, using a topic guide that had been developed with reference to NPT[14] covering: (i) introduction of e-consultations into the practice; views, promotion, training needed, (ii) e-consultation processing, (iii) impact on workload, (iv) impact on clinical practice, and (v) attitudes to future implementation. All interviews were audio recorded, transcribed, anonymised, checked for accuracy and imported into NVivo 10 software to aid analysis. Inductive thematic analysis was used grounded in the data,[33] NPT was then used as a framework to order the codes. Analysis was conducted by two researchers (MF and JB) with a subset double coded to ensure rigour. Data collection and analysis was conducted in parallel, with participants sampled until data saturation was reached. Key analytic themes were discussed within the research team to enhance credibility and external validity.

Patient record data

Electronic anonymised patient record data were collected from a random sample of patients (n=485) who had used e-consultations from eight of the participating practices (Table 1), between April 2015 to June 2016. A staff member from a participating practice retrospectively extracted anonymised patient data from patient records onto an Excel database from all practices, including: patient demographics; reason for contact; the actions taken resulting from the e-consultation (e.g. telephone call, face-to-face appointment, email advice); and further care provided by the practice in the next 30 days in relation to the e-consultation (e.g. treatment room tests, nurse appointments, further GP consultations etc.). When analysing practice responses to e-consultations, the primary response was designated as the most resource-intensive action (i.e. a face to face appointment is more resource intensive than a phone appointment than a prescription), and the secondary action was the next most resource intensive (i.e. a prescription or advice) to account for multiple e-consultation processes.[30] The primary clinical reason for patients using an e-consultation was cross tabulated with the primary response to the e-consultation from practice staff using descriptive statistics.

Patient survey data

Patient survey data were routinely collated by the e-consultation software company [Hurley], using their own survey design. Patients who opted in were sent a questionnaire seven days after the submission of their e-consultation. This contained both tick box questions and free text. We were given access to this anonymised data from the software company for the 36 pilot GP practices from April 2015 to June 2016. The tick box questions were analysed using descriptive statistics (Supplementary file, Table A). Free text comments were coded using inductive thematic analysis

For peer review only - http://bmjopen.bmj.com/site/about/guidelines.xhtml

grounded in the data,[33] NPT was then used as a framework to order the codes. Answers were analysed by two researchers (MF and JB), with a subset double coded. Patient survey quotes are labelled P01, P02… in the following data analysis. From 7,472 e-consultations, a total of 751 patients (10%) submitted a survey with quantitative data, and additional comments to individual questions ranged from 38 to 512 patients (Supplementary file, Tables A-B). Qualitative patient survey data was used to facilitate interpretation of the quantitative patient survey responses.

Theoretical integration of patient and staff data using NPT and co-production theory

Service co-production theory and NPT were theoretically integrated to examine not only implementation from staff and patient's points of view, but also the processes and interactions between patients and staff when using the e-consultation system. Service co-production can be understood as a process where service quality is shaped by (a) people's initial *expectations* of a service (b) staff and service users' *roles, interactions and experiences* within a service, leading to (c) their *resulting satisfaction or dissatisfaction.*[23 29 34] Understanding this process helps to analyse service users' roles as a co-producer of a service.[26 35] NPT constructs [14 16] and service co-production processes[23] can be integrated together and used to analyse staff and patients' initial expectations, interactions with and experiences of e-consultations, and their subsequent perceptions resulting in satisfaction/ dissatisfaction (Figure 2).

Figure 2: Combining NPT framework with service co-production processes

Patient survey data (quantitative and qualitative), staff interview data and patient record data were theoretically integrated,[36] bringing different findings together into this theoretically-informed framework (Figure 2). The NPT concepts of *coherence* and *cognitive participation* were analysed using staff interviews and patients' survey responses. *Coherence* explored staff and patients' expectations of the system and how the system's purpose and possibilities were understood. *Cognitive participation* explored the relational work that promoted engagement with e-consultations. *Collective action* explored how the system was operationalised. Initially an e-consultation workflow process map for each practice was developed from staff interviews and practice protocols on using the system. These were integrated to illustrate core practice processes. 'Touchpoints' (points of contact and interaction through a service process) were identified by using service blueprint techniques to map the e-consultation process.[25 37] Service blueprints are maps of service systems that illustrate service user and staff roles, actions and interactions, and can illustrate how service users expectations and experiences affects service quality.[25 37] Using staff interviews of the e-consultation process, and qualitative patient survey responses, three 'touchpoints'[25 35] were

For peer review only - http://bmjopen.bmj.com/site/about/guidelines.xhtml

identified, where patients and staff interacted through the e-consultation process. Touchpoints have been used in co-production literature[25 35] and health service improvement methods such as experience based co-design.[38-40] Here they were used as an analytic lens to examine the operational work and experiences of both staff and patients through an e-consultation. Key touchpoints are illustrated in Figure 3 and analysed in the *Collective action* results section.

Reflexive monitoring explored staff and patient appraisal of the system, analysing when e-consultations may work for whom. Patients' clinical reasons for using an e-consultation and practice staff responses from patient record data (Table 2) were integrated with the analysis of qualitative staff and patients' comments about their satisfaction with the system, integrating all data sets. This integration of qualitative and quantitative data used established 'following a thread'[36] techniques where the question of why staff and patients were satisfied/ dissatisfied with the system, was traced using all data sets, to understand patients and staff sources of satisfaction/dissatisfaction with the system.

RESULTS

The results are presented using the four NPT concepts, as detailed above.

Coherence

Coherence describes patients' and staff understandings and expectations of the system's purpose. E-consultations were seen by practice staff as a new way of working that had the potential to reduce GP workload pressures:

> *We are massively overstretched … So, that was one of the reasons why I wanted [eConsult], was so that we could make it easier… to deal with queries and often relatively simple queries that come through* (PM23).

Practices were aware of the difficulties patients faced in securing GP appointments and e-consultations were seen to provide a different pathway to care and advice. The pilot provided practice managers with an opportunity to test out the system without financial investment.

Patients saw e-consultations as a new, alternative way to communicate with their practice, that could be used out of surgery hours, *'It is quick and easy to use at a time to suit myself. Saves having to call the surgery'* (P61). Several patients' comments exhibited an understanding of the pressures that practices were under: '*It saves the GP time, saves me coming to the practice, great all round'* (P81).

For peer review only - http://bmjopen.bmj.com/site/about/guidelines.xhtml

Cognitive participation

Cognitive participation describes the relational work that people were involved in to promote participation with the system. Implementing e-consultations within practices was reported by practice staff as a relatively straight forward process, with little training needed. However, there were varying feelings toward it:

> *We were quite happy to do it* (AD08).

> *I was feeling very anxious about the extra workload … some things feel like a bottomless pit* (GP22).

Few practices reported involving patients in implementing e-consultations, one practice mentioned their patient participation group were concerned the system may disadvantage those who were less able to use technology. Practices employed different promotion methods to patients to varying degrees including through their website, waiting room banners, leaflets, social media, on phone answering messages, and newsletters. In some practices, there was a feeling that there was not as much uptake of the system as expected.

Some patients were activated to use the system because they couldn't get an appointment: *'No available appointment for 2 weeks'* (P10); *'Tried Switchboard nine times … Went online'* (P05). Others favoured the online format and remote consultation style, they used the system as it was difficult to visit the practice due to disabilities, illness or working commitments, or saw it was a more legitimate way to access GP advice: *'didn't want to waste Drs time with a full consultation which I didn't need'* (P171).

Collective action

Collective action describes how the system was operationalised in practice by patients and staff. Figure 3 maps where staff and patients interacted through an e-consultation process, identifying three touchpoints, key interactions and experiences through the co-production of an e-consultation.

Figure 3: E-consultation process map highlighting key touchpoints

Touchpoint 1: Patient interaction with e-consultation system

Touchpoint 1 in Figure 3 relates to patients' initial decision to complete an e-consultation form, and their interaction with the system. Over the 15 month pilot period, 7,472 patients completed an 'e-consultation', most frequently on weekdays and during traditional working hours.[30] Patient record data shows that women used e-consultations more than men (64.7% versus 35.3%) and 53.4% were

For peer review only - http://bmjopen.bmj.com/site/about/guidelines.xhtml

between 25-44 years old. [30] Most commonly, patients submitted administrative requests e.g. repeat prescriptions, test results and letters (22.5%), followed by immunological/ infection issues (14.4%) (see Table 2 and Edwards et al.[30]). Most patient survey respondents agreed that the system was easy to use (Supplementary file, Table A): *'had no problems at all'* (P398). It was '*helpful to be able to contact about minor requests*' (P475). Some patients preferred the written interface over a verbal conversation: '*Allowed me time to describe symptoms in greater detail than talking'* (P279). However, patients reported that the system did not seem to account for multiple conditions.

Touchpoint 2: GP Practice processing of e-consultations

During the pilot, the system was not integrated with the patient record IT system [EMIS] that practices used. Administrators downloaded patients' e-consultations from the system and printed them or manually imported them into patients' records. Some administrators spoke of conducting some triage e.g. directing hay fever queries to pharmacy. Clinicians described variability in the quality of information from the e-consultation forms. Whilst information could be: '*clear and concise'* (GP13), this was not always the case:

> *One patient needed to be admitted [to hospital] ... Because the symptoms weren't very clear* (GP05).

Most GPs often reverted to face to face or phone conversations to gain more information to conduct clinical decision-making.[30 31] One clinician who had substantial experience of conducting phone triage, reported that they dealt with most e-consultations without needing to see patients face-to-face, unless it was for new acute symptoms/ diagnosis.

Touchpoint 3: GP Practice interaction with patients following their e-consultation

Practices organised follow-up appointments in different ways. In some, a face to face appointment might have to *'start from scratch'* (GP05), because a different clinician originally dealt with the e-consultation:

> *I had to repeat everything I entered on line. What's the point in asking if you're not going to read it?* (P90)

Other practices had more continuity where GPs could follow through the e-consultation, which provided benefits to the consultation:

> *The actual face to face consultation is then quicker, and that's quite nice in some ways …it doesn't open up other avenues, to a degree, okay, so it's more efficient* (GP18)

For peer review only - http://bmjopen.bmj.com/site/about/guidelines.xhtml

Reception staff usually contacted patients via a practice email address or phone, to relay a message from a GP to patients, or to arrange the next step or outcome of their e-consultation. Occasionally, patients who had had no opportunity to speak to a doctor, were unhappy about this: '*I had no opportunity to ask any questions*' (P44). Some patients reported missing practice phone calls, one spoke of '*telephone answer machine ping-pong*' (P275). 14% of survey respondents reported not being contacted at all following their e-consultation (surveys were sent seven days after an e-consultation submission) which left patients dissatisfied (Supplementary file, Table A):

> *I feel like my treatment has been compromised and delayed as a result of this service* (P48)

The system had an in-built function to electronically respond to a patient's email address; however only one out of six practices where interviews were conducted said they used this, and not all staff could access the system.

Table 2 cross tabulates the primary clinical reason for patients using an e-consultation with the primary response to the e-consultation from practice staff. GP responses varied with patients' health queries e.g. medication queries and advice resulted in no face-to-face appointments, whilst 54% of neurological queries resulted in face-to-face appointments.

Table 2: Primary response from practice staff by reason for e-consultation (from patient record data)

**Patient reason for consulting**		**GP practice staff response to e-consultation**							
	**Total number (%)**	**Face to face**	**Phone consult**	**Prescription**	**Fit note**	**Test request**	**Refer routine**	**Advice**	**Other/unknown**
**Musculoskeletal / limb pain**	60 (12.4%)	48.3%	38.3%	1.7%	0	1.7%	3.3%	1.7%	0
**Infection/ Immunological**	70 (14.4%)	40.0%	41.4%	8.6%	0	0	0	0	0
**Neurological**	26 (5.4%)	53.9%	26.9%	0	0	3.9%	0	0	3.9%
**Sexual/Reproductive health**	41 (8.5%)	39.0%	41.5%	7.3%	0	4.9%	0	0	2.4%
**Dermatological**	33 (6.8%)	48.5%	21.2%	18.2%	0	0	0	3.0%	0
**Respiratory**	25 (5.1%)	52.0%	24.0%	4.0%	0	0	0	0	8.0%
**Mental health**	29 (5.9%)	44.8%	34.5%	10.3%	0	0	0	0	0
**Digestive**	19 (3.9%)	52.6%	26.3%	5.3%	0	0	0	0	5.3%

For peer review only - http://bmjopen.bmj.com/site/about/guidelines.xhtml

**Medication query/advice**	19 (3.9%)	0	73.7%	10.5%	0	0	0	0	5.3%
**Administrative** [a]	109 (22.5%)	12.2%	27.1%	11.2%	14.0%	1.9%	5.6%	10.3%	7.5%
**Other / Unclear**	54 (11.1%)	38.4%	17.0%	0	0	3.8%	0	5.7%	1.9%
**Total**	**485 (100%)**	**38.1%**	**32.1%**	**7.2%**	**3.1%**	**1.6%**	**1.6%**	**9.1%**	**6.4%**

[a] fit notes, test results, referrals, repeat scripts, letter requests, booking appointments

Patient record data in Table 2 illustrates that overall, 38% of e-consultations resulted in a primary response of a face to face consultation.[30] Several patients commented that they had received easier access to a face to face appointment through the system:

> *The service recommended immediate attention that resulted in a quicker appointment than otherwise would have been the case* (P313)

Whilst a face to face consultation often satisfied patients, it could potentially duplicate GP workload,[31] with initial e-consultation processing by administrators and a GP, plus an appointment space. 32% of e-consultations resulted in a primary response of a phone consultation. Where e-consultations resulted in a primary response of a prescription (7.2%), a 'fit note' statement of fitness for work (3.1%), test or treatment request (1.6%), referral (1.6%) or advice (9.1%) (occurring in 23% of patient e-consultation records),[30] these could save GP time as administrative staff relayed messages and there was no direct contact between the patient and GP.

> *It helps in terms of administratively if there are things which can be done very simply, and that can free up, that can free up surgery time, to a degree* (GP18).

Reflexive monitoring: Who do e-consultations work for, and when?

Reflexive monitoring describes how patients and staff appraised the system and their resulting satisfaction/ dissatisfaction. All data sets are integrated to explore when e-consultations were likely to work best for whom.

Patients' satisfaction with the system was high and most (81%) were likely to recommend the system to others. 76% said they would use the service again instead of booking a face to face appointment (Supplementary file, Table A). Dissatisfaction with the system was usually a result of: lack of interaction with a GP; missed communications; thinking that their query could be answered remotely, and then being asked to book an appointment; or lack of timely follow-up of their e-consultation. Several patients suggested improvements (at touchpoint 1) that have since been integrated into the system by the software developers e.g. allowing patients to consult with multiple symptoms for both new and existing conditions; the ability to upload photos; being able to nominate

For peer review only - http://bmjopen.bmj.com/site/about/guidelines.xhtml

a preferred GP; simplification of language; [41] and an administration channel for requests such as a fit note or test results.

Interviews revealed that clinical staff were less satisfied with the system, as time saved in completing e-consultations without further GP-patient communication (23%), was counterbalanced by e-consultation processing and GPs needing to phone or see patients in 70% of e-consultations, which could duplicate GP work.[31] Analysing why patients were e-consulting and the resulting action (Table 2), and combining this with staff and patients' appraisal of the system, Table 3 summarises when e-consultations were likely to work and be effective for patients and GPs. For GPs, it was only for relatively straightforward queries that the system could save substantial time. Patients were satisfied more often as e-consultations could: save them time, get them a quicker appointment, provide an easier access route to GP services, or they preferred the remote access format.

Table 3: Nature of e-consultations and the resulting possible satisfaction and dissatisfaction of staff and patients

Nature of query	Patients' satisfaction	Practice staff satisfaction
Administrative queries	✓<br>Most processed remotely	✓<br>Most processed remotely
Medication queries and simple queries about pre-existing patient conditions	✓<br>Most processed remotely by phone or prescription	✓<br>Most processed remotely by phone or prescription
Queries about new conditions	✓<br>May get quicker response	X<br>Face to face appointment more likely - possible work duplication
Complex questions, multiple symptoms	✓<br>May get quicker response	X<br>Face to face appointment more likely - possible work duplication

Practice suggestions for system improvement (at touchpoint 1) included that patients could be signposted away from consulting a GP more often, to encourage more self-help or use of pharmacy when '*appropriate*' (AD11), to '*make people aware that they're in some cases wasting GP's time*' (AD04). Some practice staff suggested that patients might be guided to use e-consultations under certain conditions where only remote GP input was likely to be needed (as in Table 3). In contrast, if a patient had multiple symptoms for a new condition for which a face to face appointment was likely to be needed, GPs suggested that a modified system could flag this, directing patients not to submit

For peer review only - http://bmjopen.bmj.com/site/about/guidelines.xhtml

an e-consultation but to directly book a face to face appointment, to avoid GP work duplication. Some practice staff were also concerned that the system might exacerbate inequalities of access for people with literacy difficulties or whose first language is not English, and those with difficulties in using a computer or mobile device.

Comparatively analysing different practice processing of e-consultations (touchpoint 2) suggests that administrative allocation of e-consultations to GPs could affect process efficiency. If administrators allocated e-consultations to a GP who had previously seen the patient (especially about similar symptoms/ conditions) this may support more efficient processing, as GPs would be more familiar with the patient and condition:

> *We like to look at each patient's notes to find out which doctor perhaps has seen this patient for that particular problem and then we would know where to direct that e-consultation* (AD09)

Administrators could also book face to face or phone consultations with the GP who had processed the e-consultation and was familiar with the patient query. This could focus the appointment, and avoid situations where patients felt that GPs appeared not to have read their e-consultation.

Improvements at touchpoint 3 (practice interaction with patients about e-consultations) include more robust practice communication mechanisms to reduce patient dissatisfaction about practice communication relating to their e-consultation. This could be supported by integration with electronic practice IT systems,[17] and further use of electronic communications back to patients that more staff can access and use.

Summarising this touchpoint analysis, highlights potential improvements to the system and its implementation (Table 4).

Table 4: Suggested improvements to implement the e-consultation system

Issues identified with Touchpoint 1: Patient decides to fill in an e-consultation form	Suggested technological improvements
Patients suggested several ways to improve system usability, such as: allowing patients to consult with multiple symptoms for both new and existing conditions; the ability to upload photos; being able to nominate a preferred GP; simplifying language, and an administration channel for requests such as a fit note or test results	Software developers have implemented these improvements to the system in its ongoing development[41]
Practice staff suggested that the system could	Better signposting to pharmacy and self-help

For peer review only - http://bmjopen.bmj.com/site/about/guidelines.xhtml

encourage more use of pharmacy or self-help options where appropriate	options on website interface
Promoting patients to use e-consultations for simple conditions and questions to save face to face appointments	Appropriate patient signposting on when to complete an e-consultation
Reducing patient e-consultation usage when they need a diagnosis about new, complex and multiple symptoms	Appropriate patient signposting on when not to complete an e-consultation but to directly book a face to face appointment to save practice staff work duplication
Reducing the use of the e-consultation system to directly access face-to-face appointments	Signposting to discourage patient use of the system if they want a face-to-face appointment
**Issues identified with Touchpoint 2: Practice processing of e-consultations**	**Suggested practice implementation improvements**
Some GPs received e-consultations that could have been dealt with by a pharmacy	Administrative triage where appropriate e.g. directing hay fever queries to pharmacy, to save GP time
Supporting more efficient processing of e-consultations, and potentially reduce follow-on face-to-face consultations	Allocate e-consultations to GPs who are familiar with the patient and their symptoms, where appropriate
**Issues identified with Touchpoint 3: Practice interaction with patients about e-consultations**	**Suggested improvements**
Patient complaints that they had to repeat information in consultation as GPs appeared not to have read e-consultations	Allocate follow-on phone and face-to-face appointments to GPs who initially process e-consultations
Patients missing or not receiving communication back from the practice about their e-consultation	More robust internal practice systems to ensure that patients receive communication back about their e-consultations<br>Stronger e-consultation and practice IT integration to support electronic communications back to patients that more practice staff can access and use

None of the 36 practices took up the system after the pilot, which would have involved paying market prices for the software. However, 13 practices were interested in continuing to use the system if costs were paid for by alternative funding sources, and technological interoperability with electronic patient record systems was further developed.

For peer review only - http://bmjopen.bmj.com/site/about/guidelines.xhtml

DISCUSSION

Key findings

Practices were originally interested in the system to improve access and create efficiencies. Whilst some patients used the system to try and save time for both themselves and their GPs, other patients were activated to use e-consultations when they could not get a timely appointment. Because practices were dependent upon patients deciding how and when to use e-consultations, clearer guidance may be needed for patients to support more efficient use of e-consultations (see Table 4, touchpoint 1).

Our findings highlight the difficulties in substituting real time interaction with an asynchronous technological interface (touchpoints 2 and 3). This could reduce professionals' ability to use tacit knowledge of patients concerns, patients' ability to negotiate treatment options and shared decision-making. GPs often needed further information when processing e-consultations, leading to face-to-face and phone consultations, which could duplicate workload. However, the system was being piloted, which meant that GPs were developing their skills in e-consultations, so phone and face-to-face consultations may decrease over time. GPs speculative fears about the perceived risks to patients of online consultations and the potential increases in workload[2 10] are to some extent causally linked through this study. For more efficient implementation of e-consultations, further consideration may be needed of when it is appropriate to use technology e.g. for less complex tasks,[9] and when face-to-face interaction is essential, such as in the diagnosis of complex symptoms[42]

Other interventions designed to improve efficiency and access in primary care highlight potential workload issues; e.g. nurse-led telephone triage may reduce GP contact time, but increase overall clinician contact time.[43] Previous e-health studies that use NPT highlight barriers of adverse effects on workload[44] and poor interactional workability of technology which can impede adoption within primary care.[45] E-consultations supported efficiencies for straightforward GP queries, but less so complex ones, showing that how patients use technology can affect its implementation.[46] Our results align with other studies that highlight potential barriers to technological implementation including that: the clinical data the system was designed to generate from patients was sometimes incomplete;[47] the system was not fully interoperable with other IT systems, and costs prohibited long-term usage.[17]

NPT and service co-production theory

Service co-production theory and touchpoints can extend NPT through focussing on how technologies change the service process and interactions between patients and staff. Whilst

For peer review only - http://bmjopen.bmj.com/site/about/guidelines.xhtml

involving patients voluntarily in co-designing technology may improve e-health technology;[17 40] this paper's contribution illustrates how service co-production theory can support the analysis of how patients co-implement technology through everyday service interactions, rather than voluntarily being involved in co-designing a service. Service co-production particularly extends the *collective action* aspects of NPT, exploring in-depth how both staff and patients operationalise and relate through a service system. Touchpoint analysis illustrates how patients and staff responded to digital prompts and interacted through the e-consultation process. This fills a research gap to specifically examine how e-health services affect clinical interactions with patients.[44 46] It shows how e-health implementation may be reconfigured through staff and service user produced knowledge[44] to improve technology and its implementation. This may tackle barriers to technological adoption, such as understanding how technology impacts care delivery, relationships between care givers and receivers, the role of patients in implementation, and how to maintain and improve ongoing implementation.[17 46]

Policy and practice implications

Technology is often promoted to improve NHS efficiency,[48] but benefits are often more limited due to implementation difficulties.[17] In this study, no practices experienced sufficient workload savings to warrant practices own financial investment in the system at current market prices, however the system did improve access for some patient groups. NHS England has offered financial support for practices to adopt online consultations.[6] Our research affirms that clear implementation guidance is needed[49] and provides recommendations to support the technological developments of e-consultations and future implementation to alleviate additional GP workload whilst improving patient access. NHS England case studies of e-consultation systems include their potential role to triage most patients.[50 51] Whilst our study gave no statistical evidence that patient socioeconomic factors affected usage rates,[30] practitioners in our qualitative study had concerns about the system's potential impact on equality of access. Further research is needed to investigate equity of access when implementing e-consultations.

Strengths and limitations

This study is one of the largest UK pilot independent evaluations of e-consultation systems to date, covering a wide range of GP practices. The broad sample of practice staff interviewed, combined with patient record data and patient survey data allows a comprehensive insight into the e-consultation system. Patients' qualitative survey comments varied in depth, but provided a wide breadth of responses, e.g. 510 respondents explained reasons for satisfaction/ dissatisfaction with the system. An early internal research report shared with the e-consultation software developers,

For peer review only - http://bmjopen.bmj.com/site/about/guidelines.xhtml

has supported improvements to the e-consultation system studied.[41] Theoretically, combining NPT and co-production theory has enabled the integration of staff's and patients' perspectives; and touchpoint analysis has suggested further improvements that can be developed. However, because the study was based on a pilot period of one online consultation system; the issues highlighted may be a result of the system studied, rather than all online consultation systems. Patient surveys were only sent to patients who had submitted an e-consultation (of which 10% responded), thus representing a self-selecting sample of those who had invested time into the system. Surveys were sent to patients seven days after they had submitted an e-consultation, which may have been before their e-consultations had been processed with 14% of patients waiting to hear back. Because e-consultation usage was low,[30] those patients using the system may be unrepresentative of the wider patient population.

CONCLUSIONS

E-consultations can increase patient access and satisfaction, but in their current form, were not perceived as creating sufficient workload efficiencies for continued practice usage. Patients' use of e-consultations impacted upon staff's appraisal of the system. Where both patients and staff interact with healthcare technology, it is in effect 'co-implemented'. Extending NPT through service co-production theory and touchpoints enables an analytic focus on service processes and interactions between staff and patients, and how the e-consultation system affected these. Mapping the co-production of an e-consultation through touchpoints[35 37] has highlighted where the system may be redesigned or implementation improved. This analysis can support more effective implementation of appropriate technology that accounts for professional and patient experiences.

FOOTNOTES

Acknowledgements

Authors thank staff from participating practices for assisting with the collection of individual-level data, and the software developers for providing web usage statistics and anonymous patient survey data.

Funding

This research is funded by the One Care Consortium Ltd and the National Institute for Health Research (NIHR) Collaboration for Leadership in Applied Health Research and Care West at University Hospitals Bristol NHS Foundation Trust. The One Care Consortium facilitated data collection. The views expressed are those of the authors and not necessarily those of the NHS, the NIHR or the Department of Health.

For peer review only - http://bmjopen.bmj.com/site/about/guidelines.xhtml

Competing interests

None declared.

Ethical approval

The study was reviewed by the NHS Health Research Authority (project ID: 204925) and ethically reviewed by the University of Bristol, Faculty of Health Sciences, Research Ethics Committee (Application 32961).

Contributors

KN, JH, HE, MF, JB, and EB were responsible for the study design and collection of data. KN, JH and EB were responsible for study management and co-ordination. CS was a project advisor throughout. MF, JB, HE and KN analysed the data. MF drafted the paper. All authors read, commented on and approved the final manuscript.

Data sharing statement

The datasets analysed during the current study are not publicly available, as participants were not asked to consent to this at the time of data collection. Related patient survey results are available in the Supplementary file.

Supplementary file

Table A: Patient survey – how and why accessed e-consultations, and resulting satisfaction

Table B: E-consultation patient survey free text response numbers

REFERENCES

1. Hobbs FDR, Bankhead C, Mukhtar T, et al. Clinical workload in UK primary care: a retrospective analysis of 100 million consultations in England, 2007-2014. *Lancet* 2016;387(10035):2323-30. doi: 10.1016/S0140-6736(16)00620-6
2. BMA. National survey of GPs: the future of general practice. London: British Medical Association 2015.
3. Kaffash J. Average waiting time for GP appointment increases 30% in a year 2016 [updated 10 June 2016. Available from: http://www.pulsetoday.co.uk/your-practice/access/average-waiting-time-for-gp-appointment-increases-30-in-a-year/20032025.article.
4. Atherton H, Ziebland S. What do we need to consider when planning, implementing and researching the use of alternatives to face-to-face consultations in primary healthcare? *Digit Health* 2016;2:2055207616675559. doi: doi:10.1177/2055207616675559
5. NHSEngland. GP Access Fund: NHS England; 2013 [Accessed 17/01/2017]. Available from: https://www.england.nhs.uk/gp/gpfv/redesign/improving-access/gp-access-fund/ accessed 17 Jan 2017.
6. NHSEngland. General Practice Forward View. NHS England, 2016.
7. NHSEngland. Online Consultation Systems: NHS England; 2016 [Available from: https://www.england.nhs.uk/gp/gpfv/redesign/gpdp/consultation-systems/ accessed 10 Feb 2017.

For peer review only - http://bmjopen.bmj.com/site/about/guidelines.xhtml

8. Hanna L, May C, Fairhurst K. Non-face-to-face consultations and communications in primary care: the role and perspective of general practice managers in Scotland. *Informatics in primary care* 2011;19(1):17-24.
9. Hanna L, May C, Fairhurst K. The place of information and communication technology-mediated consultations in primary care: GPs' perspectives. *Family practice* 2012;29(3):361-6. doi: 10.1093/fampra/cmr087 [published Online First: 2011/10/19]
10. Brant H, Atherton H, Ziebland S, et al. Using alternatives to face-to-face consultations: a survey of prevalence and attitudes in general practice. *The British journal of general practice : the journal of the Royal College of General Practitioners* 2016 doi: 10.3399/bjgp16X685597
11. The One Care Consortium [Accessed 17/01/2017]. Available from: http://onecareconsortium.co.uk/about/pmcf-programme/ accessed 17 Jan 2017.
12. EMISHealth. eConsult: A platform that enables patients to self-manage and consult online: Emis Health; 2016 [Accessed 30/06/2016]. Available from: https://www.emishealth.com/products/econsult/ accessed 30 Jun 2016
13. Maddan A. webGP: the virtual general practice. Pilot report. London: Hurley Group, 2014.
14. May C, Finch T. Implementing, Embedding, and Integrating Practices: An Outline of Normalization Process Theory. *Sociology* 2009;43(3):535-54. doi: 10.1177/0038038509103208
15. Murray E, Treweek S, Pope C, et al. Normalisation process theory: a framework for developing, evaluating and implementing complex interventions. *BMC Med* 2010;8(1):1-11. doi: 10.1186/1741-7015-8-63
16. May C, Rapley T, Mair FS, et al. Normalization Process Theory On-line Users' Manual, Toolkit and NoMAD instrument. 2015 [Available from: http://www.normalizationprocess.org.
17. Ross J, Stevenson F, Lau R, et al. Factors that influence the implementation of e-health: a systematic review of systematic reviews (an update). *Implement Sci* 2016;11(1):146. doi: 10.1186/s13012-016-0510-7
18. Gibson J, Lightbody E, McLoughlin A, et al. 'It was like he was in the room with us': patients' and carers' perspectives of telemedicine in acute stroke. *Health Expect* 2016;19(1):98-111. doi: 10.1111/hex.12333
19. Kennedy A, Vassilev I, James E, et al. Implementing a social network intervention designed to enhance and diversify support for people with long-term conditions. A qualitative study. *Implement Sci* 2016;11(1):1-15. doi: 10.1186/s13012-016-0384-8
20. Clarke DJ, Godfrey M, Hawkins R, et al. Implementing a training intervention to support caregivers after stroke: a process evaluation examining the initiation and embedding of programme change. *Implement Sci* 2013;8(1):96. doi: 10.1186/1748-5908-8-96
21. Connell LA, McMahon NE, Harris JE, et al. A formative evaluation of the implementation of an upper limb stroke rehabilitation intervention in clinical practice: a qualitative interview study. *Implement Sci* 2014;9(1):1-12. doi: 10.1186/s13012-014-0090-3
22. Brandsen T, Honingh M. Distinguishing Different Types of Coproduction: A Conceptual Analysis Based on the Classical Definitions. *Publ Admin Rev* 2016;76(3):427-35. doi: 10.1111/puar.12465
23. Osborne SP, Radnor Z, Strokosch K. Co-Production and the Co-Creation of Value in Public Services: A suitable case for treatment? *Public Manag Rev* 2016;18(5):639-53. doi: 10.1080/14719037.2015.1111927
24. Batalden M, Batalden P, Margolis P, et al. Coproduction of healthcare service. *BMJ Qual Saf* 2015 doi: 10.1136/bmjqs-2015-004315
25. Radnor Z, Osborne SP, Kinder T, et al. Operationalizing Co-Production in Public Services Delivery: The contribution of service blueprinting. *Public Manag Rev* 2014;16(3):402-23. doi: 10.1080/14719037.2013.848923
26. Alford J. Co-Production, Interdependence and Publicness: Extending public service-dominant logic. *Public Manag Rev* 2016;18(5):673-91. doi: 10.1080/14719037.2015.1111659

For peer review only - http://bmjopen.bmj.com/site/about/guidelines.xhtml

27. Ordanini A, Pasini P. Service co-production and value co-creation: The case for a service-oriented architecture (SOA). *EMJ* 2008;26(5):289-97. doi: http://dx.doi.org/10.1016/j.emj.2008.04.005
28. Osborne SP, Radnor Z, Nasi G. A New Theory for Public Service Management? Toward a (Public) Service-Dominant Approach. *ARPA* 2013;43(2):135-58. doi: 10.1177/0275074012466935
29. Osborne SP, Strokosch K. It takes Two to Tango? Understanding the Co-production of Public Services by Integrating the Services Management and Public Administration Perspectives. *British Journal of Management* 2013;24:S31-S47. doi: 10.1111/1467-8551.12010
30. Edwards H, Marques E, Hollingworth W, et al. Use of a primary care online consultation system, by whom, when and why: evaluation of a pilot observational study in 36 general practices in South West England. *BMJ Open* 2017;7:e016901 doi: 10.1136/bmjopen-2017-016901
31. Banks J, Farr M, Salisbury C, et al. The use of an electronic consultation system in primary care: views and experiences from general practice. *Br J Gen Pract.*2017 doi: 10.3399/bjgp17X693509
32. PublicHealthEngland. National General Practice Profiles 2017 [Available from: https://fingertips.phe.org.uk/profile/general-practice/data accessed 22 Nov 2017.
33. Braun V, Clarke V. Using thematic analysis in psychology. *Qualitative Research in Psychology* 2006;3:77-101.
34. Parasuraman A, Zeithaml VA, Berry LL. A Conceptual Model of Service Quality and Its Implications for Future Research. *J Mark* 1985;49(4):41-50. doi: 10.2307/1251430
35. Parker S, Heapy J. The Journey to the Interface: How public service design can connect users to reform. London: Demos, 2006.
36. Moran-Ellis J, Alexander VD, Cronin A, et al. Triangulation and integration: processes, claims and implications. *Qualitative Research* 2006;6(1):45-59. doi: 10.1177/1468794106058870
37. Bitner MJ, Ostrom AL, Morgan FN. Service Blueprinting: A practical technique for service innovation. *Calif Manage Rev* 2008;50(3):66-94.
38. Bate P, Robert G. Bringing user experience to healthcare improvement: the concepts, methods and practices of experience-based design. Oxford: Radcliffe Publishing 2007.
39. Boaz A, Robert G, Locock L, et al. What patients do and their impact on implementation. *J Health Organ Manag* 2016;30(2):258-78. doi: 10.1108/jhom-02-2015-0027 [published Online First: 2016/04/08]
40. Wherton J, Sugarhood P, Procter R, et al. Co-production in practice: how people with assisted living needs can help design and evolve technologies and services. *Implement Sci* 2015;10:75. doi: 10.1186/s13012-015-0271-8
41. Ellender M. E-consultations can work in the NHS - it's a learning curve. Response to: Electronic consultations offer few benefits for GP practices, says study. *BMJ* 2017;359 doi: 10.1136/bmj.j5141
42. Jiwa M, Meng X. Video Consultation Use by Australian General Practitioners: Video Vignette Study. *Journal of medical Internet research* 2013;15(6):e117. doi: 10.2196/jmir.2638
43. Holt TA, Fletcher E, Warren F, et al. Telephone triage systems in UK general practice: analysis of consultation duration during the index day in a pragmatic randomised controlled trial. *The British journal of general practice : the journal of the Royal College of General Practitioners* 2016;66(644):e214-e18. doi: 10.3399/bjgp16X684001
44. Mair FS, May C, O'Donnell C, et al. Factors that promote or inhibit the implementation of e-health systems: an explanatory systematic review. *Bulletin of the World Health Organization* 2012;90(5):357-64. doi: 10.2471/blt.11.099424 [published Online First: 2012/05/17]
45. Murray E, Burns J, May C, et al. Why is it difficult to implement e-health initiatives? A qualitative study. *Implementation Science* 2011;6(1):1-11. doi: 10.1186/1748-5908-6-6
46. May CR, Finch TL, Cornford J, et al. Integrating telecare for chronic disease management in the community: What needs to be done? *BMC Health Serv Res* 2011;11(1):131. doi: 10.1186/1472-6963-11-131

For peer review only - http://bmjopen.bmj.com/site/about/guidelines.xhtml

47. Greenhalgh T, Wherton J, Papoutsi C, et al. Beyond Adoption: A New Framework for Theorizing and Evaluating Nonadoption, Abandonment, and Challenges to the Scale-Up, Spread, and Sustainability of Health and Care Technologies. *Journal of medical Internet research* 2017;19(11):e367. doi: 10.2196/jmir.8775
48. Greenhalgh T, Procter R, Wherton J, et al. The organising vision for telehealth and telecare: discourse analysis. *BMJ Open* 2012;2(4) doi: 10.1136/bmjopen-2012-001574
49. Stokes-Lampard H, Stodel M, Bullard E, et al. GP Forward View: Assessment of progress Year 1. London: Royal College of General Practitioners, 2017.
50. NHSEngland. New consultation types - eConsult in Southampton NHSEngland; 2017 [Available from: https://www.england.nhs.uk/publication/10-high-impact-actions-new-types-of-consultation-econsult-in-southampton/ accessed 10 Nov 2017.
51. Varnam R. Online consultations at Unity Health, York: NHS Networks; 2017 [Available from: https://www.networks.nhs.uk/nhs-networks/releasing-capacity-in-general-practice/messageboard/2-new-consultation-types/128512817 accessed 10 Nov 2017.

Figure 1: NPT constructs in association with the implementation of e-consultations

Figure 2: Combining NPT framework with service co-production processes

Figure 3: E-consultation process map highlighting key touchpoints

For peer review only

For peer review only - http://bmjopen.bmj.com/site/about/guidelines.xhtml

NPT Construct	
**Coherence**	Sense-making work to understand the possibilities of an intervention.<br>*What are the purposes of e-consultations?*
**Cognitive participation**	Relational work that builds a community of practice around an intervention.<br>*What promotes participation with e-consultations?*
**Collective action**	Operational work that people enact to make an intervention function.<br>*How do participants interact with e-consultations to make them work?*
**Reflexive monitoring**	Appraisal work where people assess how a new practice affects them and others.<br>*How do participants appraise e-consultations?*

Figure 1: NPT constructs in association with the implementation of e-consultations

148x64mm (300 x 300 DPI)

For peer review only

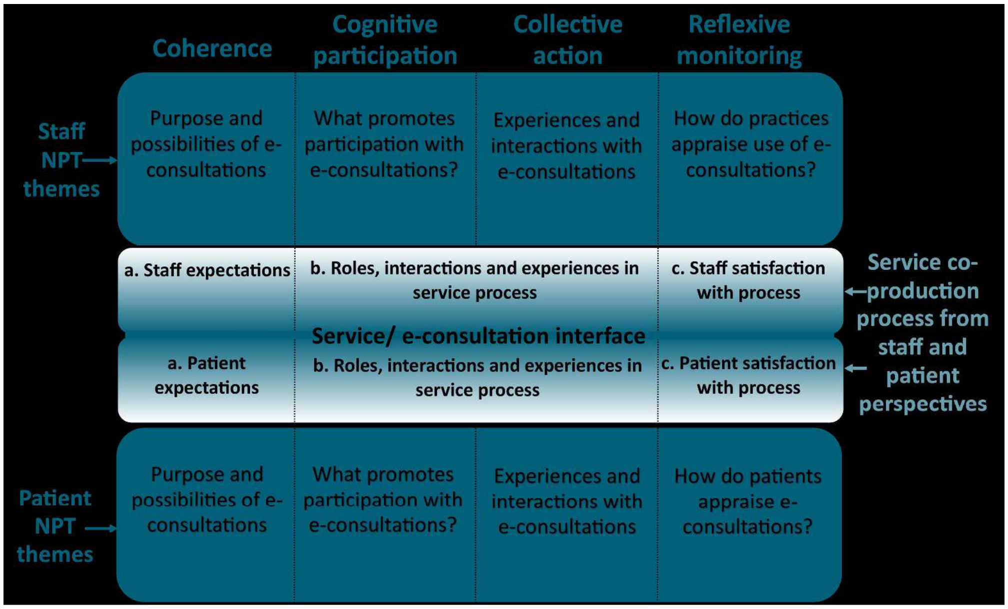

Figure 2: Combining NPT framework with service co-production processes

285x171mm (300 x 300 DPI)

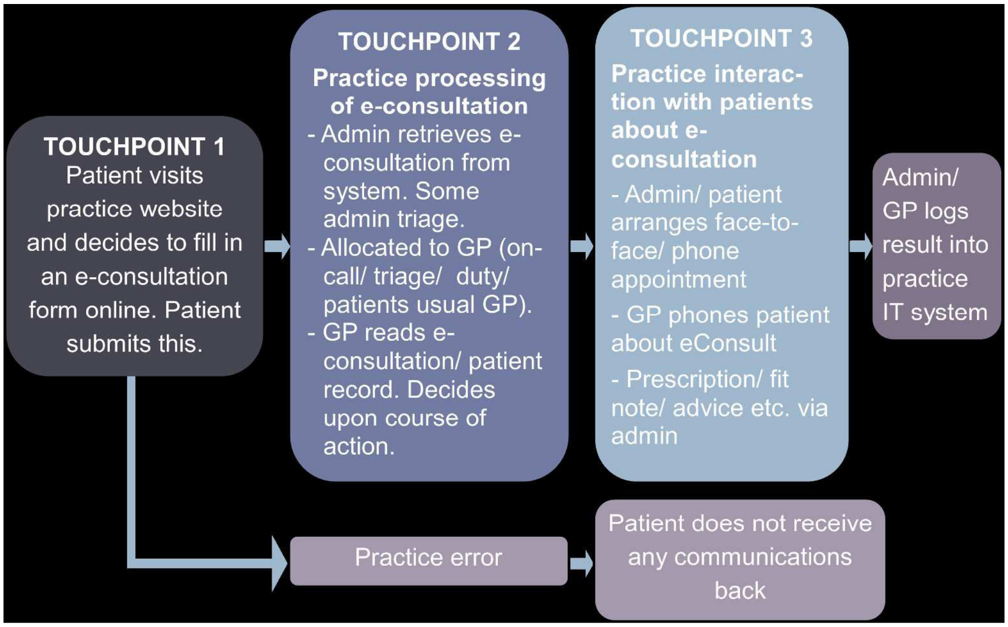

Figure 3: E-consultation process map highlighting key touchpoints

286x177mm (300 x 300 DPI)

SUPPLEMENTARY FILE

Table A: Patient survey – how and why accessed e-consultations, and resulting satisfaction

Some of these questions are expanded further with free-text responses, overviewed in Table B.

	% (n)
How did you hear about the Consult Online from Home Service?	
My GP told me about it	0.3% (2)
Someone else from the GP practice	4.2% (33)
From the GP practice website	58.0% (455)
Another patient/family member	4.2% (33)
From an internet search	5.6% (44)
I read about it	2.7% (21)
From a leaflet or promotional banner	18.5% (145)
Other → Free text	6.6% (52)
When your practice contacted you about your Consult Online From Home Service assessment, what were you advised to do?	
How to look after the problem myself, without contacting the GP practice/other health service	9.1% (56)
Pick up a prescription from the surgery /pharmacy	27.6% (169)
Visit the GP practice for face-to-face at later date	40.0% (244)
Go to A&E department / Walk-in Centre etc	1.5% (9)
Was not contacted	13.7% (84)
Other → Free text	8.3% (51)
How likely are you to recommend the Consult Online From Home Service to friends and family if they need similar care or advice?	
Extremely likely	55.5% (422)
Likely	25.2% (192)
Neither likely nor unlikely	7.2% (55)
Unlikely	6.6% (50)
Extremely unlikely	5.5% (42)
Would you use the service again instead of booking a face to face appointment?	
Yes	76.3% (582)
No	8.5% (65)
Not sure	15.2% (116)
Thinking about your use of the Consult Online From Home Service, would you agree or disagree with the statements below	
The Consult Online From Home Service was easy to use.	
Strongly agree	50.7% (383)
Agree	37.6% (284)
Neither agree nor disagree	5.2% (39)
Disagree	5.2% (39)
Strongly disagree	1.5% (11)

For peer review only

	% (n)
Overall, how satisfied or dissatisfied were you with using the Consult Online From Home Service for your health assessment?	
Very satisfied	60.3% (459)
Fairly satisfied	20.4% (155)
Neither satisfied nor dissatisfied	4.3% (33)
Fairly dissatisfied	7.4% (56)
Very dissatisfied	7.6% (58)

Table B: E-consultation patient survey free text response numbers

Table B summarises the free text questions from the eConsult online patient satisfaction survey, with response numbers. Average word counts of comments, alongside the range of comments from the lowest number of words in a comment to the highest are provided to give details on the breadth and depth of comments

E-consultation survey question	Number of free text responses	Average word count of comments	Word count range of comments
How did you hear about the Consult Online?	49	6.3	2 to 18
If the Consult Online service had not been available, what would you have done about your health problem?	37	11.4	1 to 67
Was there a practical reason why you used the Consult Online?	182	12.9	1 to 68
When your practice contacted you about your Consult Online assessment, what were you advised to do?	224	10.8	1 to 93
Did you follow the Consult Online advice? (if answered no, reason why they did not follow advice)	74	8.6	1 to 27
Did any of our staff make your experience particularly good?	343	16.7	1 to 187
Overall, how satisfied or dissatisfied were you with using the Consult Online Service for your health assessment? Reason for this	510	28.1	1 to 257
What improvements would you make to the service?	512	16.3	1 to 127

For peer review only

COREQ Statement

Implementing online consultations in primary care: A mixed method evaluation extending normalisation process theory through service co-production

Domain 1: Research team and reflexivity

Personal Characteristics

1. Interviewer/facilitator. Which author/s conducted the interview or focus group?

Jon Banks (JB)
Michelle Farr (MF)
(Included as authors, p.1, Title page)

2. Credentials. What were the researcher's credentials? E.g. PhD, MD.

JB: BA (WEngland), PhD (Wales), PGdip (Wales)
MF: BSc (Hons), MPhil, PhD, FHEA
(page number not applicable)

3. Occupation. What was their occupation at the time of the study?

JB: Research Fellow: Applied Social Science (Qualitative), National Institute for Health Research, Collaborations for Leadership in Applied Health Research and Care West (NIHR CLAHRC West), University of Bristol

MF: Senior Research Associate in Applied Social Science (Qualitative) Research, National Institute for Health Research, Collaborations for Leadership in Applied Health Research and Care West (NIHR CLAHRC West)
(institutional affiliations given, p.1)

4. Gender. Was the researcher male or female?

JB: Male
MF: Female.
(page number not applicable)

5. Experience and training. What experience or training did the researcher have?

JB: Post graduate diploma in social science research methods as part of PhD, experience of collecting and analysing qualitative data with 15 years as a research associate and research fellow.
MF: Extensive training in qualitative research methods (MPhil, PhD), taught qualitative research methods to undergraduates. 12 years' experience conducting qualitative research.
(page number not applicable)

Relationship with participants

6. Relationship established. Was a relationship established prior to study commencement?

No relationship was established before the commencement of study (p.5 gives details of how practices and participants were sampled).

7. Participant knowledge of the interviewer. What did the participants know about the researcher? e.g. personal goals, reasons for doing the research?

The professional goals of the researchers were to complete the aims and objectives of the study only. The researchers had no personal goals or reasons for doing the research. As part of recruitment and gaining informed consent clinicians were fully informed about the aims and objectives of the study through participant information sheets (p.5).

For peer review only

8. Interviewer characteristics. What characteristics were reported about the interviewer/facilitator? e.g. Bias, assumptions, reasons and interests in the research topic.

The interviewers (JB and MF) were both social scientists.

All participants were aware that the interviews were for independent academic research through participant information sheets (p.5).

The researchers had no personal interests in the study, it was solely their professional role.

Domain 2: study design

Theoretical framework

9. Methodological orientation and Theory. What methodological orientation was stated to underpin the study? e.g. grounded theory, discourse analysis, ethnography, phenomenology, content analysis

In the Background section we explain how we use normalisation process theory and service co-production theory to understand how both patients and staff co-implement and use technology (p.4).

In the Methods section we explain how NPT and co-production theory were used to integrate staff and patient perspectives on e-consultations (p.7 Heading: Using NPT and co-production to integrate patient and staff perspectives).

Participant selection

10. Sampling. How were participants selected? e.g. purposive, convenience, consecutive, snowball.

GP practices were purposively sampled to ensure a range of: locations (rural/suburban/urban); practice levels of deprivation measured by the Index of Multiple Deprivation (IMD) from practice postcodes; and volume of e-consultation usage (calculated by dividing the number of e-consultations received by the number of days the system was live at time of sampling). Six practices were purposively sampled to conduct qualitative interviews. A purposive sample of staff with different professional roles from these six practices involved in the processing or managing of e-consultations were invited to be interviewed via email, with contacts and invitations facilitated by practice managers. Please see Methods section Sampling and Recruitment (p.5) and Table 2.

11. Method of approach. How were participants approached? e.g. face-to-face, telephone, mail, email

Six practices were sampled to be invited to take part in the qualitative research, with six replacements with a similar profile if any of the first six did not want to participate. Practices were initially approached and invited to take part in the research by OneCare staff who were the study collaborators and, as a GP consortium, had direct contact with the practices. OneCare staff phoned practices and emailed them a letter to explain about the research and what getting involved would mean in terms of time and resources. If practices were interested in taking part and wanted to be contacted by the researchers, OneCare staff gave the researchers the practice managers email and telephone number to arrange a discussion about getting involved in the research. Once practice managers had agreed to take part in the research they identified who may be appropriate staff to be interviewed, focusing on those who were involved in the e-consultation system. Practice managers initially approached GPs and administrative staff to ask if they were interested in participating in research interviews. All interviewees were given participant information sheets either via the practice manager, and/ or by researchers. Interviews were organised with different staff via the practice manager. Before interviews commenced, researchers went through the participant information sheet with participants, with further opportunities to ask questions about the research. See Heading: Sampling and recruitment (p.5).

12. Sample size. How many participants were in the study?

We interviewed 23 practice staff. Please see Table 2: Sampled GP practice and interview participant profiles (p.5-6).

For peer review only

13. Non-participation. How many people refused to participate or dropped out? Reasons
Two practices did not want to take part, so we invited those reserve practices that had a similar profile to the ones who did not want to take part. Both two reserve practices agreed to take part. Because practice managers spoke to practice staff initially about taking part in interviews (p.5) we do not know how many practice staff declined to take part in an interview, as we only spoke to those who were willing to take part.

Setting
14. Setting of data collection. Where was the data collected? e.g. home, clinic, workplace
Interviews took place both face to face within general practice offices (n=20) and over the phone (n=3) (p.6).

15. Presence of non-participants. Was anyone else present besides the participants and researchers?
Most interviews took place in private offices, with just the interviewee. However, two of the practice manager interviews and two of the interviews with administrative staff were held in shared office spaces but as this was about work based processes we do not believe that this inhibited the interviews in any way (page number not applicable).

16. Description of sample. What are the important characteristics of the sample? e.g. demographic data, date.
Interviews were conducted with 23 practice staff including: 10 GPs, 1 nurse practitioner (NP) (this interviewee is designated with (GP) notation to avoid potential identification), 6 practice managers (PM), 6 'administrators' including an IT manager and receptionists (AD). Please see Table 2: Sampled GP practice and interview participant profiles and Heading: Qualitative interviews and analysis (p.5-6).

Data collection
17. Interview guide. Were questions, prompts, guides provided by the authors? Was it pilot tested?
A semi-structured, topic guide was used for all interviews, guided by NPT. This was discussed and agreed with OneCare staff, the research partners (Heading: Qualitative interviews and analysis, p.6).

18. Repeat interviews.
We did not conduct repeat interviews (p.5-6 gives the list of interviews and interviewees).

19. Audio/visual recording. Did the research use audio or visual recording to collect the data?
Yes audio recording was used through interview data collection, with full informed consent (Heading: Qualitative interviews and analysis, p.6).

20. Field notes. Were field notes made during and/or after the interview or focus group?
Yes, some summary notes were made in addition to the voice recorded interviews (page number not applicable).

21. Duration. What was the duration of the interviews or focus group?
Interviews lasted between 10-40 minutes (Heading: Qualitative interviews and analysis, p.6).

22. Data saturation. Was data saturation discussed?
Data saturation was discussed in research management meetings. Data collection and analysis was conducted in parallel, with participants sampled until data saturation was reached (Heading: Qualitative interviews and analysis, p.6).

For peer review only

23. Transcripts returned. Were transcripts returned to participants for comment and/or correction?
No. We did not feel this was possible to offer in the time available in the study (page number not applicable).

Domain 3: analysis and findings

Data analysis

24. Number of data coders. How many data coders coded the data?
Analysis was conducted by two researchers (MF and JB) with a subset double coded to ensure rigour. Key themes were discussed within the research team to enhance credibility and external validity (Heading: Qualitative interviews and analysis, p.6).

25. Description of the coding tree. Did authors provide a description of the coding tree?
The coding tree is not included in the manuscript due to word limits (page number not applicable).

26. Derivation of themes. Were themes identified in advance or derived from the data?
Themes were derived inductively from the data. These were then ordered using NPT as a framework (Heading: Qualitative interviews and analysis, p.6 and Heading: Using NPT and co-production to integrate patient and staff perspectives, p.7).

27. Software. What software, if applicable, was used to manage the data?
We used NVivo 10 qualitative software package to manage the data (Heading: Qualitative interviews and analysis, p.6).

28. Participant checking. Did participants provide feedback on the findings?
Participants did not provide feedback on the initial findings. However emerging research themes were discussed in research management meetings with OneCare, as research collaborators (page number not applicable).

29. Quotations presented. Were participant quotations presented to illustrate the themes / findings? Was each quotation identified? e.g. participant number
Yes unique participant codes are used alongside quotes, to illustrate findings. The professional roles of the interviewees were identified within the codes. (Heading: Results, p.8-14)

30. Data and findings consistent. Was there consistency between the data presented and the findings?
Yes (Heading: Results, p.8-14)

31. Clarity of major themes. Were major themes clearly presented in the findings?
Yes (Heading: Results, p.8-14)

32. Clarity of minor themes. Is there a description of diverse cases or discussion of minor themes?
Yes a range of practitioner perspectives are included (Heading: Results, p.8-14). For example, one clinician who had substantial experience of conducting phone triage, reported that they dealt with most e-consultations without needing to see patients face-to-face, unless it was for new acute symptoms/ diagnosis. This potentially suggests that GPs may get more used to this consultation approach over time (p.10).

For peer review only
